# Spatial analysis, local people’s perception and economic valuation of wetland ecosystem services in the Usumacinta floodplain, Southern Mexico

**DOI:** 10.7717/peerj.8395

**Published:** 2020-01-31

**Authors:** Vera Camacho-Valdez, Andrea Saenz-Arroyo, Andrea Ghermandi, Dario A. Navarrete-Gutiérrez, Rocío Rodiles-Hernández

**Affiliations:** 1 Departamento de Conservación de la Biodiversidad, CONACYT-El Colegio de la Frontera Sur, San Cristóbal de las Casas, Chiapas, Mexico; 2 Departamento de Conservación de la Biodiversidad, El Colegio de la Frontera Sur, San Cristóbal de las Casas, Chiapas, Mexico; 3 Department of Natural Resources and Environmental Management, University of Haifa, Haifa, Haifa, Israel; 4 Laboratorio de Análisis de Información Geográfica y Estadística, El Colegio de la Frontera Sur, San Cristóbal de las Casas, Chiapas, Mexico

**Keywords:** Usumacinta floodplain, Ecosystem services, Wetland, Local perception, Livelihoods, Spatial analysis

## Abstract

The Usumacinta floodplain is an exceptional area for biodiversity with important ecosystem services for local people. The main objective of this paper was to estimate reference values and define local perceptions of ecosystem services provided by wetlands and overlapping them with spatially explicit socioeconomic and biodiversity indicators. We used the Usumacinta floodplain as an example of a territory where high dependence of rural people on ecosystem services is confronted with development projects that threat the flow of ecosystem services, thus affecting rural people well-being. With a combination of data from remote sensing, global databases of ecosystem service values, local perception of ecosystem services and socioeconomic and biodiversity richness indicators in a spatially explicit framework, we develop a policy-oriented approach for rapid assessment to manage wetlands and maintain people’s livelihoods. Regulating and provisioning services are identified as the most relevant ecosystem services in terms of their monetary value and local perceived importance. In a spatially explicit manner, this approach highlights the most valuable wetlands and identifies rural societies that are highly dependent on ecosystem services. Our approach can be replicated elsewhere and could provide valuable information for policymakers to design policies that can contribute to conserve wetland ecosystems where under threat of development.

## Introduction

The need to accommodate both the challenges of improved environmental conservation as well as maintaining cultural diversity and wellbeing of vulnerable sectors of the population has played a central role in the development of the ecosystem services framework and conservation discourse (Daily, 1997;  MEA, 2005; Durand, 2019). In this context, wetland ecosystems, such as those occurring in floodplains, have been the subject of numerous ecosystem service assessments and valuations worldwide (Kanyiginya et al., 2010; Rongoei et al., 2013; Richards et al., 2015; Ghermandi et al., 2010), due to the crucial role they play in supporting the livelihoods of local populations through their provisioning (e.g., fibre, water, food, fuel wood, natural medicines), regulating (e.g., flood control, water quality improvement), and cultural services (e.g., support of recreation, aesthetic value, support of educational activities) (TEEB in Policy, 2011).

In spite of their social and ecological importance, floodplain wetlands are threatened worldwide mainly due to land use conversion for economic activities such as agriculture, tourism, urban development, forestry and fossil energy production (Renaud et al., 2013; Day et al., 2016). For example, a global overview of dam-based impacts on large river systems shows that over half are affected by dams, thereby resulting in the alteration of functional processes and diminishing of the provision of ecosystem services to local societies (Nilsson et al., 2005). From an economic perspective, such alterations can be seen as the outcome of the undervaluation of the public goods and services provided by these ecosystems in decision-making processes regarding their use, management and conservation (Brander et al., 2012).

The Usumacinta floodplain, a 700,000 ha wetland ecosystem in Southern Mexico which was selected as case-study site for the present research, is representative of the above mentioned global trend. Since the 1980s, the discovery of a large hydrocarbon deposit on the marine platform near the floodplain has led to major land use changes in the region, making the oil industry one of the main economic activities in the area (Yáñez-Arancibia, Day & Currie-Alder, 2009). This development, together with other activities such as intensive agriculture and stockbreeding, has increased the exploitation and deterioration of the wetland ecosystem, declining the provision of ecosystem services to local communities (García-Cuéllar et al., 2004; García García & Kauffer Michel, 2011). Although there have been governmental efforts to protect the ecosystems by declaring two important wetlands within the floodplain as natural protected areas (Centla Swamps and Terminos Lagoon, respectively in 1992 and 1994), a recent spatial analysis shows that this area—in particular the Centla Swamps Biosphere Reserve - has experienced dramatic transformations in the last 20 years, from natural floodplain vegetation to cattle and agricultural areas (De la Rosa-Velázquez et al., 2017; Ghermandi et al., 2010) which carries with economic difference such as the concentration of benefits as well as environmental and social problems (Durand, 2019). In order to reverse this trend and guarantee the access of communities to environmental resources and the preservation of sustainable livelihoods, more democratic local policy interventions are needed (Sundberg, 2003) to steer these processes and mitigate their negative impacts on ecosystems and society in general (Adams et al., 2004). An analysis of the relationship between cultural identity and conservation is essential to dealing with the environmental issues (Sundberg, 2006).

Since policies targeting biodiversity conservation may have important social implications, it is necessary to develop assessments that include relevant a range of social, ecological and economic aspects (Schmidt, Sachse & Walz, 2016; Ciftcioglu, 2017) as well as local people’s perspective of what is important to preserve. In this regard, the ecosystem service framework may help to understand user preferences and relative values placed on ecosystem services (De Groot et al., 2012). In turn, this provides important support information for policy-makers and stakeholders to make informed decisions involving wetland resource allocation when faced with competing uses (Chaikumbung, Doucouliagos & Scarborough, 2016) as well as to implement more integrative and effective conservation strategies that recognize the needs of local people and the economic costs of private or public interventions (Camacho-Valdez et al., 2013; Adusumilli, 2015).

Despite the emphasis that is placed in the literature on the importance of integrating the social, ecological, and economic aspects in the evaluation of ecosystem services, most empirical studies in the international arena rely on assessments derived from single disciplinary approaches (Gómez-Baggethun et al., 2014). In particular, while the need is recognized to complement instrumental perspectives often based on monetary-based approaches to the assessment of ecosystem service values with a more pluralistic approach explicitly embracing, among others, socio-cultural and biophysical measures (IPBES, 2016), a unified theoretical framework is still lacking. There is a need for more integrative empirical studies combining tools and assessment methods from different disciplines. This is arguably particularly true in rural and remote areas where the linkage between ecosystem services and human well-being occurs at finer spatial scales (Wu, 2013). One should note that such pluralistic valuation approach does not entail a rejection of monetary valuation techniques per se, but rather emphasizes the benefits of integrating them with the complementary information that can be obtained through a broad suite of valuation methods (Bernues et al., 2014; Wam et al., 2016; Jacobs et al., 2016).

In recent years, spatial analysis through Geographic Information Systems (GIS) has been increasingly relied upon in the investigation and representation of the ecosystem services provided by different ecosystems at a range of different scales (Naidoo et al., 2008; Luck, Chan & Fay, 2009; Lautenbach et al., 2011; Lavorel & Grigulis, 2012; Ghermandi, Ding & Nunes, 2013). Spatially explicit assessments can be helpful for instance in analyzing trade-offs and synergies among ecosystem services in a particular landscape (Raudsepp-Hearne, Peterson & Bennett, 2010). In this context, ecosystem services mapping is becoming an important tool for land-use planning and conservation-related decision-making (Burkhard & Maes, 2017).

This paper integrates spatial analysis with results from different ecosystem service assessment methods relying both on monetary valuation techniques and participatory, socio-cultural valuation techniques applied in the local communities to provide useful, spatially explicit insights on the provision of wetland ecosystem services in the Usumacinta floodplain, with a particular focus on producing valuable information for the local environmental decision makers. More specifically, we aim at identifying important wetland ecosystem services through the combination of different monetary and non-monetary valuation techniques and combining such information with indicators of biodiversity and socioeconomic marginalization to identify priority areas for wetland conservation. First, a spatial analysis of the wetland types that are present in the Usumacinta floodplain is performed, taking advantage of the use of remote sensing and GIS. Through the application of value transfer techniques, reference monetary values for different ecosystem services provided by the identified wetland types were calculated. We contrasted these results with the perspective of local inhabitants with respect to wetland ecosystems services in four communities located in the study area. The study finally explores the spatial variability of the estimated ecosystem service values as they overlap with spatially explicit indicators of the socioeconomic marginalization of the local population and biodiversity richness, to provide a multidimensional perspective on priority areas for natural capital conservation within the study area.

### Study area

**Figure 1:**
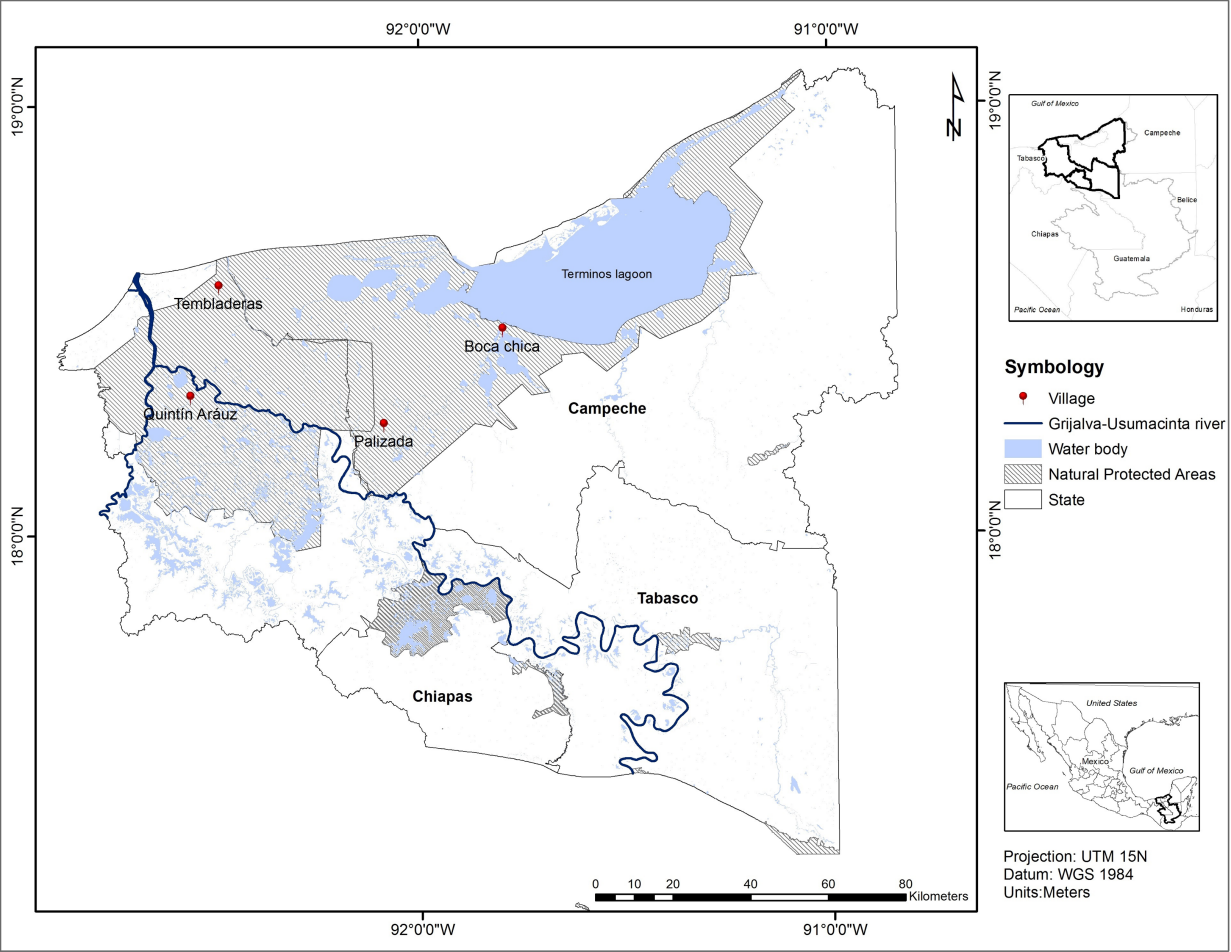
Study area. Usumacinta floodplain (UFP), southern Mexico. Location of surveyed villages. The layers are derived from INEGI open data.

The study site has a surface of approximately 2.5 million ha and is located between the states of Campeche, Tabasco and Chiapas, in southern Mexico. The floodplain is part of the Usumacinta watershed and comprises a main river, the Usumacinta, a major tributary, the Grijalva River, and the Terminos Lagoon (Fig. 1). Over the past 50 years, four large dams have been built along the Grijalva River causing significant impact on the surrounding landscape, watershed fragmentation and changes in the natural water flow regulation (Wilkerson, 1986; Muñoz Salinas & Castillo, 2015). The main physical factors that affect the Usumacinta floodplain wetlands are: (a) precipitation, (b) flood pulses, and (c) the coastal plain of Tabasco and Campeche. Freshwater pulses with high suspended sediments, inorganic nutrients and organic materials generate extensive wetlands such as Terminos Lagoon and Centla Swamps, the latter being the largest coastal lowland wetland in Mesoamerica (Yáñez-Arancibia, Day & Currie-Alder, 2009).

Before the development of the oil industry in the 1980s, fishing was the main economic activity in this region (Tudela, 1989). The lagoon and its associated habitats serve as nursery and feeding areas for important species of shrimp and about 214 species of fish in the southern Gulf of Mexico (Lara-Domínguez, Arreguín & Álvarez Guillén, 1993). Previous studies have shown that fishery resources in Campeche Sound and Terminos Lagoon depend strongly on the supply of nutrients, organic matter, flood pulses and the movement of pre-adult fish and shrimp from the lagoon-estuarine system to the sea caused by tidal action (Deegan et al., 1986; Yañez Arancibia, Aguirre-León & Soberón-Chavez, 1992). Fisheries also include reef fish, coastal migratory pelagic fish, and large oceanic pelagics of great importance at an international level (Yáñez-Arancibia & Day, 2004), which also depend on the ecological integrity of the Usumacinta floodplain system, its waters and the quality of their habitats.

Overall, 14 municipalities are located in the study region, all showing high degrees of marginalization, with the exception of Carmen and Centro. Ciudad del Carmen is the most important city, with a population of 221,000 inhabitants and infrastructure to support the oil industry and other economic activities (e.g., tourism, fishing, harboring) (INEGI, 2010). The study area is mostly characterized by a warm, humid climate with 1,200–2,500 mm rainfall during the summer and an average annual temperature of 26–27 °C (INEGI, 2008).

## Materials & Methods

**Figure 2:**
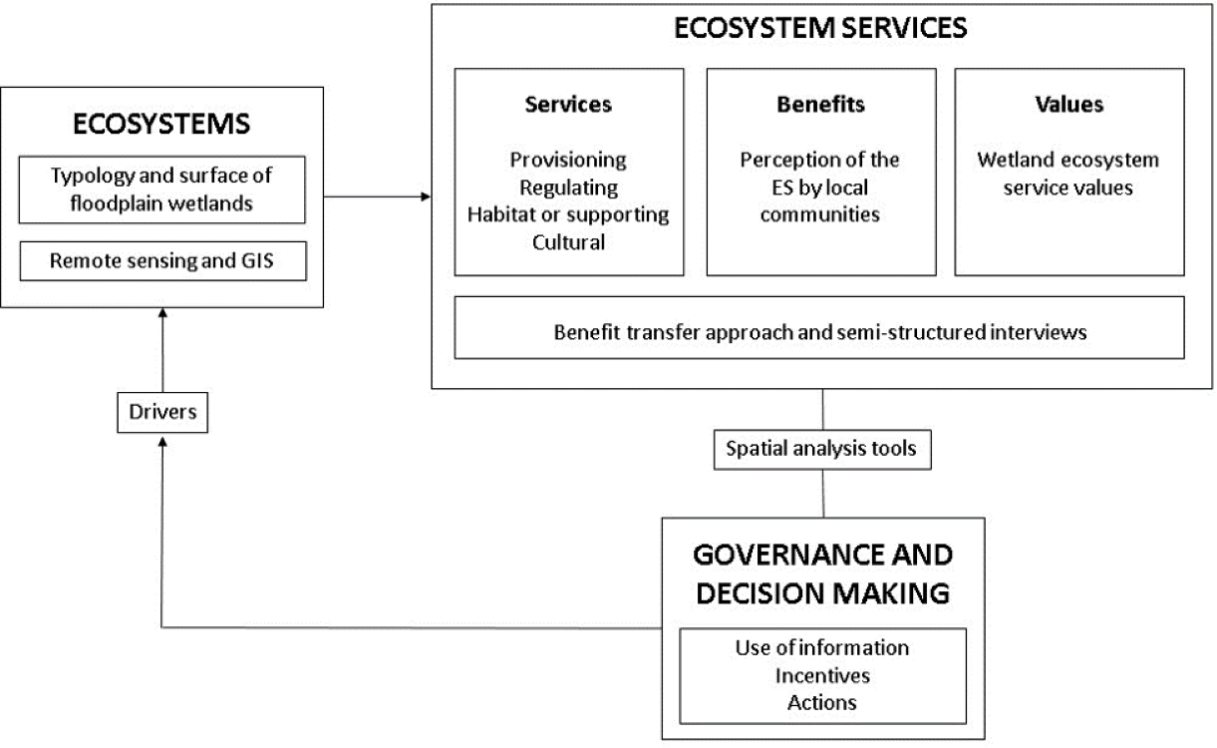
The methodological approach for ecosystem valuation in the Usumacinta floodplain (modified from TEEB in Policy (2011), Ciftcioglu (2017) and Haines-Young & Potschin (2018)).

The methodological approach used in this research draws from various disciplines and includes: (i) the classification and characterization of spatial distribution of land use and wetland ecosystem types through remote sensing and GIS techniques; (ii) the valuation of local wetland ecosystem services based both on the results of an international value transfer exercise and (iii) the analysis of the perception of ecosystem services as elicited by local inhabitants through semi-structured interviews; and (iv) an analysis of the spatial overlap of the estimated ecosystem service values with selected indicators of population marginalization and biodiversity richness in order to provide policy-relevant information for decision-makers (Fig. 2).

### Spatial analysis of wetland ecosystems distribution

Different types of floodplain wetlands, their distribution and extension were identified from the classification of Landsat 8 OLI (path /row: 21/ 47; 22/47 and path /row: 21/ 48; 22/48) images acquired in April, May and November 2014. IDRISI Selva and ArcGIS 10 were used for the classification process and for the final coverage map. Before starting the classification procedure, Landsat image was limited to the margins of the physiographic province of the Southern Gulf Coastal Plain (INEGI, 2008) using a masking process, and geographically projected to the UTM zone 15 North (WGS84). Subsequently atmospheric correction was performed using the Idrisi Selva AtmosC module. The atmospheric correction is an essential requirement to have an accurate representation in the analysis of the surface properties from satellite images (Mahdavi et al., 2018).

The classification process was carried out using a supervised method which required the user to identify the wetland types, digitizing on-line from a color composite image a sample of pixels (training sites) for each cover. Using the maximum likelihood algorithm individual pixels were assigned to the cover class with the highest degree of similarity (Campbell, 1996). For the definition of the wetland types present in the study area, the Mexican wetland classification proposed by Berlanga-Robles, Ruiz-Luna & Lanza Espino (2008) was used.

The accuracy of the final map was assessed by an error matrix and the Kappa index (K’). An error matrix was developed using 30 test points per class as reference data and compared by cross-tabulation with pixels from the classification. Coincidences between both datasets (main diagonal) were used to estimate the overall accuracy (%) and Kappa coefficient (K’) to measure the correspondence between the classification and the reference data (Congalton & Green, 1999). The test points for the analysis were randomly selected from the resulting final map and field validated with the assistance of a GPS. The accuracy for the classification was 86% and the Kappa coefficient was 0.85, indicating that the classification results meet the accuracy requirement of land cover classification.

### Wetland ecosystem services in the Usumacinta floodplain

#### Ecosystem services valuation

Although previous research recommends the application of primary valuation techniques as the preferred approach to the monetary valuation of ecosystem services (Farber et al., 2006), conducting such studies at large scales is often not a viable option due to time and funding constraints, lack of the necessary infrastructure, and the inherent, logistical difficulties (Shrestha & Loomis, 2001; Rosenberger, 2015). As a second-best option, benefit transfer methods have been widely applied (Johnston et al., 2015). Such techniques rely on the application of monetary values and other information derived from the original study site(s) to a policy site (Liu et al., 2012). Some of the main limitations of benefit transfer include data availability and reliability, distribution of data on services and values over biomes, differences in socio-economic context, and spatial heterogeneity (De Groot et al., 2012). The consensus among value transfer practitioners is that the more similar the study and policy are, the more accurate the value transfer will be (Rosenberger & Phipps, 2007; Johnston & Rosenberger, 2010; Kaul et al., 2013; Rosenberger, 2015). However, the literature has yet to agree on a set of criteria for site similarity (Johnston & Rosenberger, 2010). Given the constraints of the present study as well as the general lack of suitable primary valuation studies from coastal wetlands in Mexico, whereby most available valuations focus on mangrove ecosystem services (Lara-Domínguez, Arreguín & Álvarez Guillén, 1993; Barbier & Strand, 1998; Mendoza-González et al., 2012), this study relies on an international value transfer to provide a first estimate of the multiple ecosystem services delivered by each wetland type and integrate them in further analysis. International datasets of 216 coastal wetland ecosystem service valuations (Ghermandi et al., 2010; Van der Ploeg et al., 2010) were used to infer the average values for each of the wetland types located in the case-study area. Although other wetland valuation databases have been produced in the literature (e.g., Chaikumbung, Doucouliagos & Scarborough, 2016; Camacho-Valdez et al., 2013), such datasets were selected upon considerations regarding the relevance for the investigated study region and their accessibility. To avoid double counting value estimates, studies that value two or more services or that estimate total economic value were not taken into account in the transfer. Values were presented in common monetary units (USD/ha/year), inflation-adjusted to 2007 USD (Eade & Moran, 1996; Costanza et al., 1997; Wilson, Troy & Costanza, 2004) and organized for the corresponding services within each of the wetland types. We determine the minimum, maximum, standard deviation and median of the economic values for most services in each wetland type (see Supplemental Information 1 provided as online supplementary information). The estimated total value associated with each wetland ecosystem service within the Usumacinta floodplain (in $US 2007 per year) was estimated multiplying the median per-hectare value by the total area of each wetland type, as derived from the spatial analysis. Finally, the estimated values were spatially represented across the Usumacinta floodplain, displaying the value of the annual flow of services provided by each wetland type.

#### Local perception of ecosystem services

In order to complement the ecosystem service value information derived from the international value transfer exercise and to partially account for the lack of local ecosystem service valuations, a qualitative study of ecosystem service values was implemented using a participatory approach in four typical villages distributed along the main type of wetlands of the Usumacinta floodplain region (Fig. 1). The aim of this implementation was to compare the results obtained with both methods, looking for similarities and differences, as well as better highlight the dependence of local people from wetland ecosystem services. To achieve this, we collected relevant data combining four different categories:

1. *Ecosystem services*: benefits people obtained from ecosystems, including provisioning (e.g., support of commercial fishing), regulating (water quality improvement), habitat, and cultural services (e.g., recreation) (TEEB in Policy, 2011).
2. *Capitals*: benefits which are expressed in the form of capital accumulated through the use of ecosystem services in human livelihoods (Scoones, 1998), such as human capital (e.g., health), social capital (e.g., associations) and man-made capital (e.g., income).
3. *Main activities:* productive activities of rural populations (e.g., fishing, tourism).
4. *Main threats:* threats and their possible impacts related to exogenous changes, both climatic and anthropogenic (e.g., water contamination, deforestation).

The methods applied to understand the importance of ecosystem services and its associations to local people‘s livelihoods were based on the FABE’s approach (Hare & Pahl-Wostl, 2002; Pahl-Wostl & Hare, 2004; Hare, 2011), which is generally used to build system dynamics models on local people’s dependence to natural capital. By incorporating the sustainable livelihoods approach (Scoones, 1998), the use of natural capital is understood on how is transformed in different forms of capital such as human capital, social capital, economic capital and infrastructure. Each of the interviewers made one casual loops model linking ecosystems attributes, with the benefits and threats they perceive where affecting their livelihoods as proposed by Fisher et al. (2013).

**Figure 3:**
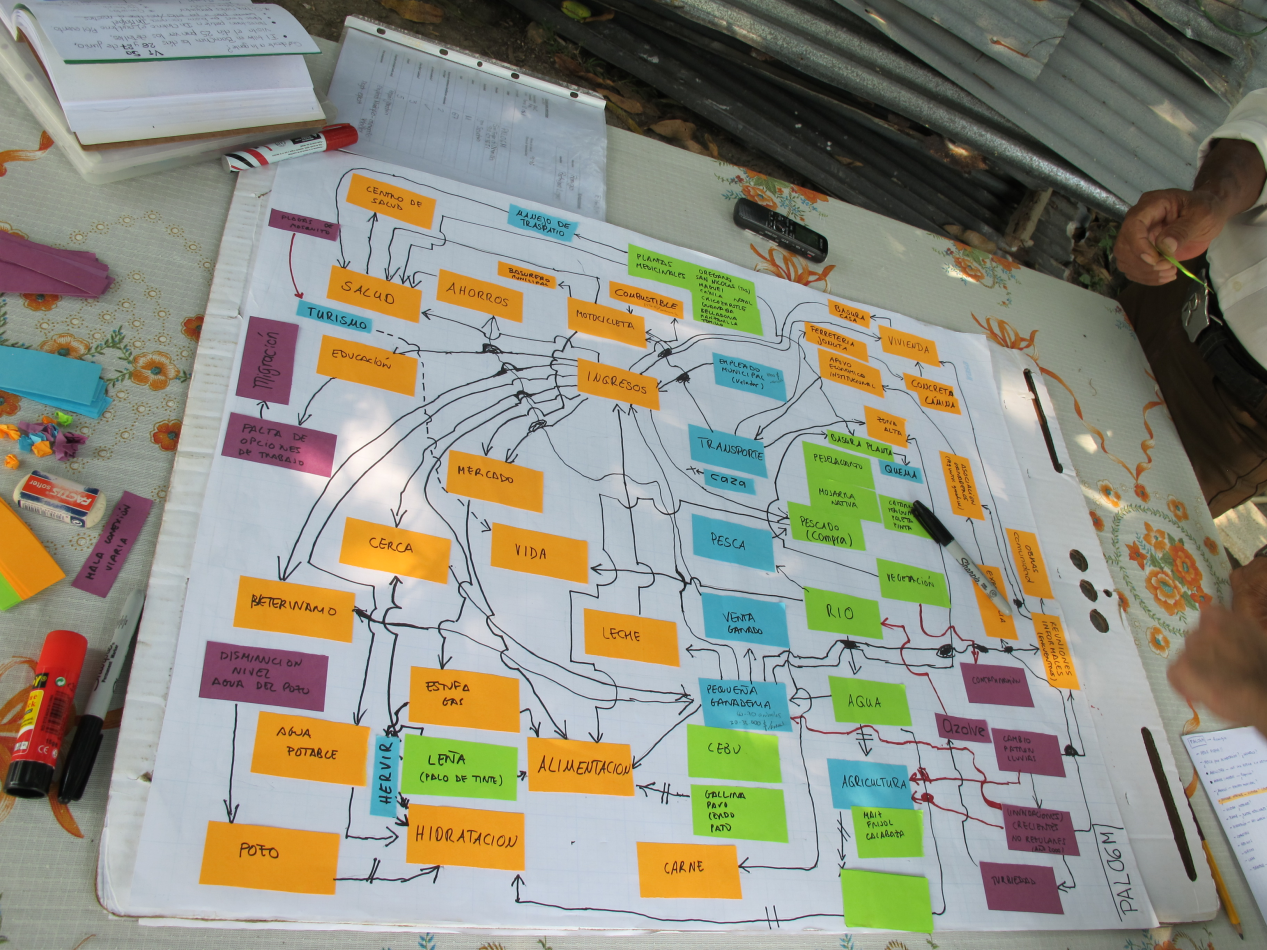
An example of a cognitive map derived from the semi-structured interview. Photo: Ellinor Roth.

Ten semi-structured in-depth interviews were conducted between October 2014 and March 2015 in each of the four communities selected (Fig. 1.). They were organized following the previously defined categories that were covered during the course of the interview, instead of a sequenced script of standardized questions. Such format for the semi-structured interviews allows for more focused, conversational, two-way communication (Meli et al., 2015). Interviews were developed around initial questions such as “What does the river mean to you?”, “What do you eat normally?”, “Where does your income come from?”, “What kinds of problems are you mostly concerned about?”. Also, in the course of each semi-structured interviews, the interviewers used the method of ”cognitive mapping” (Hare, 2011) to develop on paper, cognitive maps of individual perspectives for each of the respondents. All interviews were digitally recorded and professionally transcribed based on the “Grounded Theory” (Glaser & Strauss, 1967) and the four previously defined categories. Subsequently, an individual qualitative conceptual model for each participant was built on a computer (Fig. 3).

The selection of the communities was based on the following criteria: (1) location, (2) population, (3) type of settlement, (4) antiquity, (5) level of transformation of the environment, 6) economic activities, 7) ethnicity, (8) level of marginalization (education level), and (9) researchers’ safety (Table 1). With the support of the National Commission of Natural Protected Areas (CONANP), communities in the region that met these criteria were selected. Once the four communities were identified, the delegate of each community was contacted and with their help, ten members were chosen in each of them. In the application, care was taken that the interviews would reflect a representation of ages, gender, educational levels and social sectors in order to have a wide representation of the uses of the ecosystems. Although we acknowledge that a larger number of interviewees and communities would provide a more comprehensive perspective on local ecosystem service perceptions, a more thorough investigation was not possible within the constraints of the study. Yet, we believe the present results to be valuable in an area with severe limitations in data availability. It is stressed that this research reports only results concerning the ecosystem services identified by local communities in order to highlight the local dependence of people from wetlands. The results of the other categories will be analyzed in depth in another article that is in preparation.

**Table 1:** General characteristics of the four selected communities.

	Quintín Arauz	Tembladeras	Palizada	Boca Chica
Location	Near the main river	Near the mangroves	Near a tributary	Near the coastal lagoon
Population^a^	1,000 inhanitants	200 inhabitants	1,000 inhabitants	15 households
Type of settlement^a^	Concrete homes Paved roads Church Health center School	Precarious houses	Concrete homes Paved roads Church Health center School	Precarious houses
Seniority of the community	200 years	40 years	300 years	60 years
Level environment transformation	High	Low	High	Low
Economic activities	Fishing/livestock	Fishing	Fishing/livestock/ tourism	Fishing
Ethnicity	Chontal	Mixed	Mixed	Mixed
Education^a^	Low-medium	Low	Low-medium-high	Low

**Notes.**

a Based on INEGI (2010).

### Spatial analysis of value flows in their socioeconomic and biodiversity context

In order to spatially investigate the link between the socioeconomic status of the population, biodiversity and the elicited ecosystem service values, we used a set of indicators and GIS to superimpose the different layers of information. The number of bird species present in the Usumacinta floodplain area was used as a proxy for biodiversity richness. Birds are among the best-studied organisms worldwide and they are often considered as good ecological proxies to assess the biodiversity values of an area, even for other taxa which are difficult to sample (Prendergast et al., 1993; Kati et al., 2004; Maccherini et al., 2009; Santi et al., 2010). The bird species distribution maps produced by International & NatureServe (2014) were used to derive the number of bird species observed in the Usumacinta floodplain, rasterized at a 30-m resolution. The socioeconomic marginalization index produced by the National Population Council (Mexico) (http://www.conapo.gob.mx/es/CONAPO/Indices_de_Marginacion), estimated at the municipal level for the 14 municipalities in the Usumacinta floodplain, was used to outline economic vulnerability in the area. Following the methodology of Ghermandi, Ding & Nunes (2013), the spatial overlap of such indicators with the value of the flow of ecosystem services was explored, both at a high resolution (30-m grid cells) and aggregated at the level of municipalities. This analysis was primarily targeted at providing relevant information for the development of environmental policy instruments through the identification of priority conservation areas.

## Results

### Ecosystems classification and land cover map

**Figure 4:**
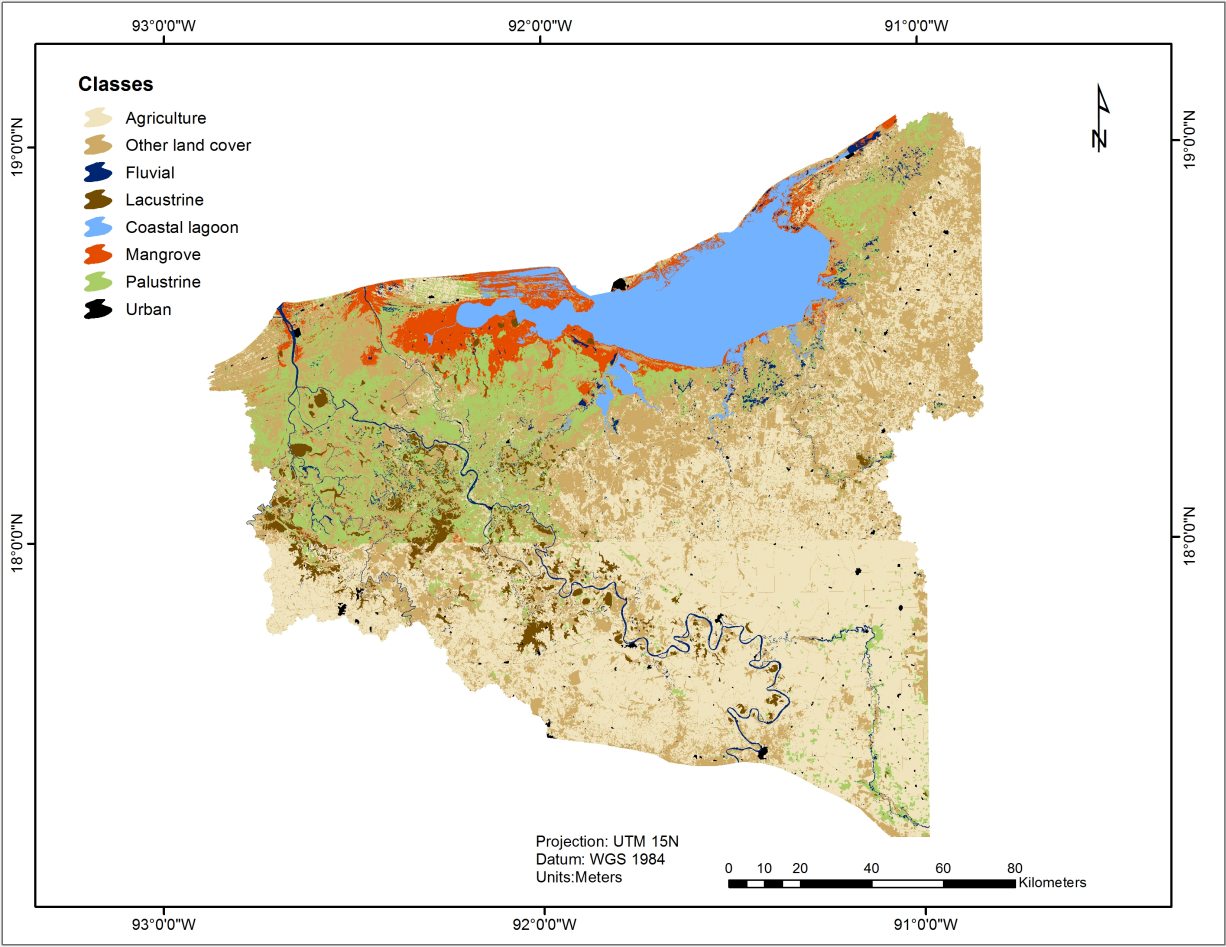
Classification of land use and land cover in the UFP from 2014 Landsat TM free imagery.

The results of the classification of wetland types and land use covers in the Usumacinta floodplain are shown in Fig. 4. The map indicates that urban areas, agriculture, wetlands (coastal lagoon, mangrove, riverine, palustrine and lacustrine) and other inland cover areas are the dominant cover types in the study area.

Considering the classification results, agriculture was the most widely represented land use (1,018,186 ha), covering 21% of the entire area. Among wetlands, the largest ecosystems are palustrine, covering 320,285 ha (12%) of the total surface in the study area. Palustrine and coastal lagoon ecosystems were the most important wetlands in terms of coverage (548,486 ha), representing 71% of the total area of wetlands. Mangroves, riverine and lacustrine ecosystems occupy substantially smaller areas (Table 2).

**Table 2:** Summary of estimated values for each service per wetland type derived from the value transfer exercise. The total ecosystem service values per wetland type obtained from adding the median values per ES (in bold). Values in 2007 USD/ha/year.

	# obs.	Mangrove	Coastal lagoon	Palustrine	Riverine	Lacustrine	Total ES median value
**Provisioning services**	**135**	**1,325**	**281**	**4,845**	**1,085**	**478**	**8,014**
Support of commercial fishing	57	380	122	67	822	446	1,838
Water supply	18	683	3	1,286	18	24	2,014
Harvesting of natural materials	39	213	156	3,353	65	8	3,794
Fuel Wood	20	48		26	180		254
Ornamental resources	1			114			114
**Regulating services**	**47**	**690**	**17**	**2,322**	**4,399**	**23**	**7,452**
Local climate control	1	7					7
Flood control and storm buffering	30	683	17	1,661	4,291	23	6,677
Water quality improvement	16			661	108		769
**Habitat or supporting services**	**21**	**247**	**0**	**55**	**2,145**	**4,369**	**6,816**
Maintenance of genetic diversity	1			31			31
Habitat for species	20	247		24	2,145	4,369	6,785
**Cultural services**	**40**	**391**	**1,629**	**2,467**	**5,202**	**1,496**	**11,185**
Amenity and esthetics	9		460		3,291	495	4,245
Recreational activities	20	362	151	2,430	1,253	896	5,093
Recreational fishing and hunting	11	28	1,018	37	658	105	1,847
Median total value per ecosystem type (2007 USD/ha/year)		2,653	1,926	9,689	12,833	6,366	
Area (ha)^a^		108,600	228,201	320,285	54,178	62,442	
Annual ecosystem service flow^b^ (2007 USD/year)		288,105,575	439,619,915	3,103,293,877	695,246,902	397,517,290	4,923,783,558^c^

**Notes.**

a Extension of the wetland in hectares.

b Annual ecosystem service flow was calculated multiplying the total ecosystem service value by the wetland area.

c Total economic value (TEV) was calculated by adding the value of the annual flow of ecosystem services by wetland type.

Wetland description. Mangrove: forested-shrub estuarine wetland: plant association formed by one or a combination of the four species of mangrove; coastal lagoon: subtidal estuarine wetland; palustrine: palustrine continental wetland (where there is permanent water): swamp, marshes, tular, popal; riverine: permanent riverine wetland: rivers and channels; and lacustrine: lacustrine continental wetland, permanent and seasonal: lake, ponds, body of water.

### Wetland ecosystem service values

#### Monetary values from benefit transfer

The results of the international value transfer analysis are presented in Table 2. The number of blank cells represents data gaps in the dataset. Overall, there is considerable variability in ecosystem service values delivered by different wetland types. On a per-hectare basis, the riverine ecosystem was the coverage with the highest annual median value of $12,833 USD/ha/year. The mangrove, palustrine and lacustrine ecosystems also contribute significantly to the valuation analysis with a median value of $2,653, $9,689 and $6,366 USD/ha/year, respectively. Coastal lagoons were the wetland type with the lowest median value of $1,926 USD/ha/year (Table 2).

The total economic values of ecosystem services show a considerable variability. Habitat for species was the ecosystem service with the highest median value ($6,785 USD/ha/year), followed by flood control and storm buffering and recreational activities ($6,677 and $5,093 USD/ha/year, respectively). In general, cultural services are the most valued (Table 2).

The value of the annual flow of ecosystem services delivered by each type of ecosystem in the Usumacinta floodplain, calculated as the product of each ecosystem service median value per area of the corresponding wetland type (Table 2), was over $4,000 million USD/year. Of all wetlands included in the analysis, the palustrine ecosystem is one that contributed the highest annual flow ($3,698 million USD/year), due to its substantial representation in the total area of the case study (12%) combined with its relatively high value ($9,689 USD/ha/year). Estuarine ecosystems (coastal lagoons and mangroves) together with the riverine ecosystem make an important contribution to the total economic value of approximately $1,000 million USD/year, while the lacustrine ecosystem made the smallest contribution ($397 million USD/year).

#### Importance of ecosystem services for local livelihoods

The analysis of semi-structured interviews allowed us to identify the dependence of local people on wetland ecosystems through the provision of ecosystem services. Respondents to the survey were all adults (>18 years) whose average age and time living in the community were 52 (range between 28 and 82 years) and 32 years, respectively; 57.5% were male and 42.5% female.

**Figure 5:**
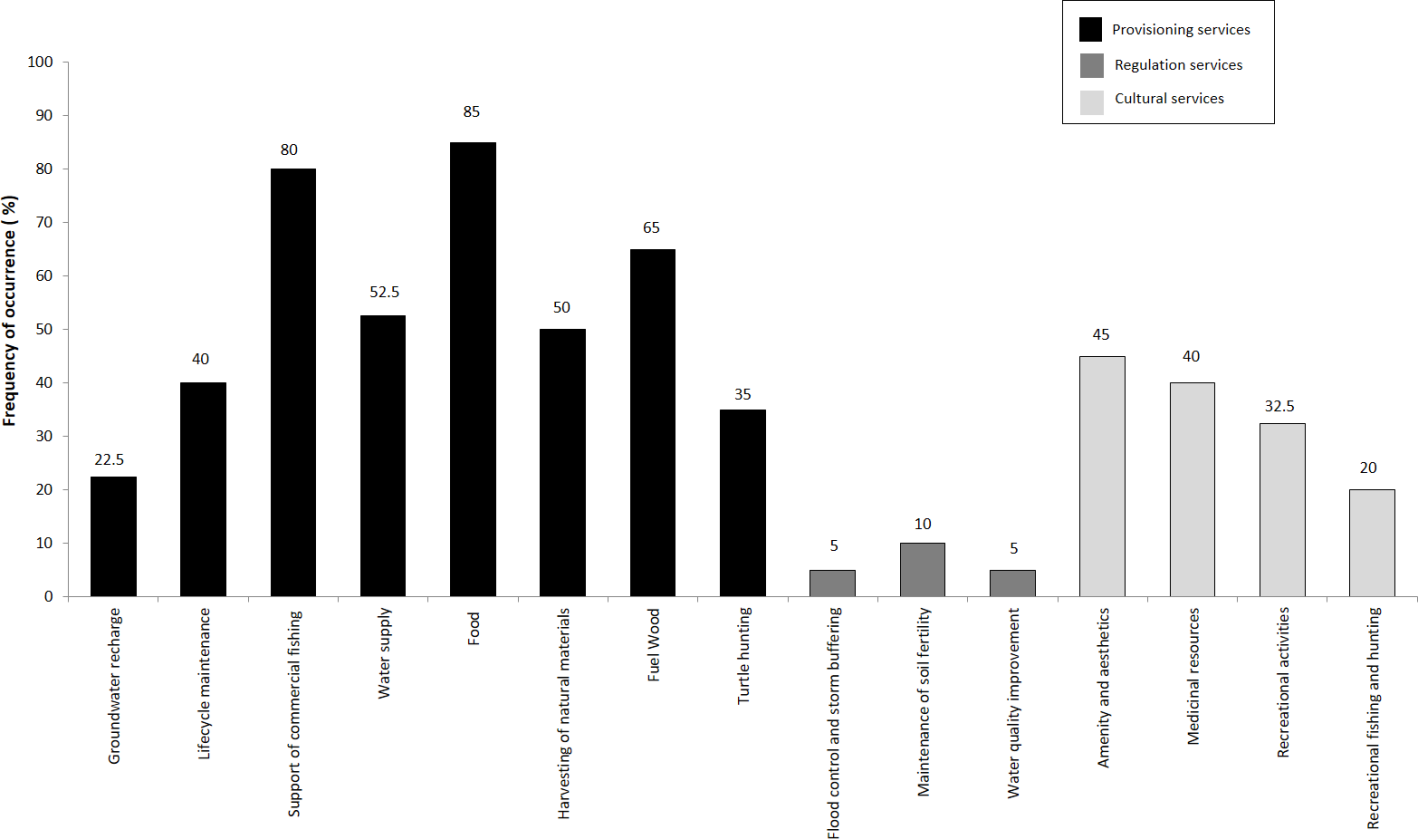
Frequency of occurrence (%) of ecosystem services in the four communities where people were interviewed. Food category includes: fish and crustaceans.

Figure 5 shows that 15 types of ecosystem services are important to local people. Among these ecosystem services, the greatest number of services mentioned in the interviews are those corresponding to the provisioning (39%), followed by cultural (26%) and finally regulating services (13%). Assessment of the provisioning services shows that the current wetlands provide diverse ecosystem services for the local people, highlighting food (85%), support of commercial fishing (80%), and fuel wood (65%). Other services frequently identified were water supply (52.5%), harvesting of natural materials (50%), amenity and aesthetics (45%), medicinal resources (40%), and recreational activities (32.5%). Flood control and storm buffering was the service least often identified by respondents (5%).

Comparing these results with the estimates obtained through the benefit transfer exercise reveals that the most valuable ecosystem services differ depending on the context. Cultural services are highlighted as the most valuable services by global studies while provisioning services are the most mentioned by local people. This differentiation may be due design of semi-structured interviews that were aimed at understanding the dependence of local communities on the ecosystem services that are most immediately tangible to them, such as of provisioning, as opposed to cultural and regulating services which are provided in an indirect way.

### Mapping the dependencies of ecosystem service values, biodiversity and socio-economic indicators

**Figure 6:**
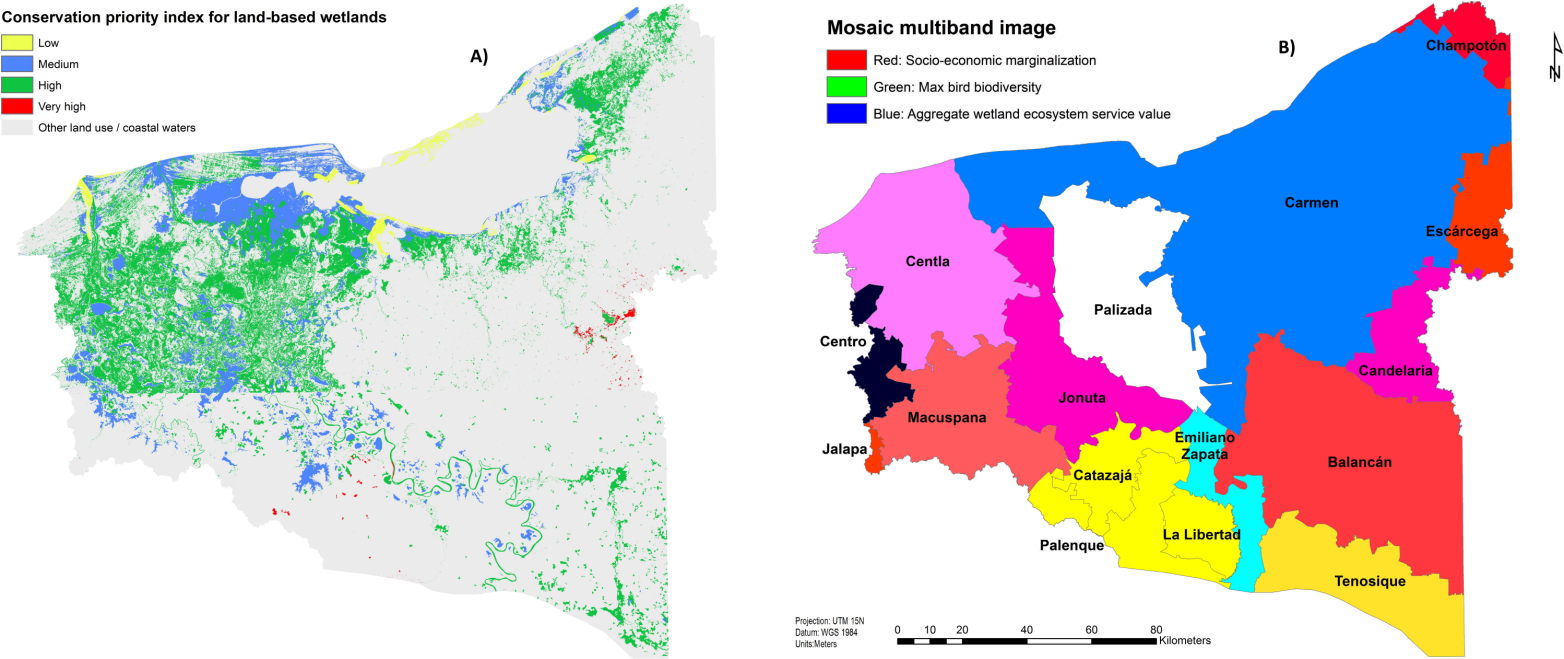
(A) Ranking of priority conservation wetland areas based on overlapping of ecosystem service values, socioeconomic and biodiversity indexes; (B) overlap of socioeconomic and biodiversity indicators with the estimated ecosystem service values in the Usumacin.

Overlapping the distribution of the estimated monetary ecosystem service values with the selected biodiversity and socio-economic indicators reveals important multi-dimensional information for the sustainable management of wetland ecosystems in the Usumacinta floodplain. Figure 6A ranks wetland grid cells (mapped at a 30-m resolution) based on wetland conservation priority. The latter is determined as follows: cells in which the values of all three individual layers are in the upper 25%ile of the respective layer are classified as very high priority; wetland cells with values of only one or two layers in the upper quartile are classified as median and high priority respectively, irrespectively as to which layers rank highly. Figure 6A shows that the majority of high priority conservation areas were concentrated in the municipalities of Palizada, Jonuta, Centla and Carmen. However, the highest degree of overlap between biodiversity, socioeconomic marginalization and ecosystem service values is observed in more isolated wetland ecosystems located in the municipalities of Candelaria and Palenque.

Given that most policy-relevant decisions are taken at the level of administrative regions, in Fig. 6B each of the municipalities in the Usumacinta floodplain is attributed a unique combination of three color bands, which reflect each of the three components of analysis: (i) blue for aggregate ecosystem service value; (ii) red for socioeconomic marginalization; and (iii) green for biodiversity richness. The values of each of the three indexes is standardized to range between 0 (minimum value) and 255 (maximum value). The saturation of the color in each band is determined by the local value of the indexes. For instance, municipalities where the ecosystem service value (e.g., Carmen) or the socioeconomic marginalization (e.g., Balancán, Jalapa, Escárcega and Champotón) components prevail over the other two are shown in blue and red colors, respectively. In yellow regions (e.g., Palenque, La Libertad and Catazajá) high biodiversity (green) and socioeconomic marginalization levels (blue) are combined with a relatively low value of the flow of ecosystem services. Municipalities with high values for each of the indexes are represented in light grey or off-white colors (e.g., Palizada). In general, Palizada municipality is depicted in white, indicating high values for each of the three indicators.

Comparing these findings with the local perception analysis highlights that the interviewed communities are distributed differently with respect to the spectrum of priority areas. For instance, Quintin Arauz and Tembladeras are located in an area of high socio-economic marginalization and high ecosystem value (magenta).

## Discussion

The present study provides a multi-dimensional perspective and spatial analysis of the provision of ecosystem services in a rural, largely marginalized region of Mexico, which (1) complements conventional economic valuation techniques with local people’s perceived importance of these services, and (2) integrates ecosystem service values into the broader context identified by the local biodiversity richness and socio-economic conditions with the purpose of providing policy guidance for the sustainable management of the rich local natural capital.

Previous analyses have demonstrated that complex relationships exist between economic development and ecosystem degradation (Das Neves Almeida et al., 2017), as well as between the provision of ecosystem services and biodiversity indicators (Naidoo et al., 2008). This implies that attention to the local conditions and the integration of insights from different approaches and disciplines is fundamental in the evaluation of different policy options and setting of conservation or development priorities.

The valuation component of the present study relies on two different approaches to elicit the contribution of different wetland ecosystem services to the well-being of the local populations. The monetary valuation results suggest that wetlands in the Usumacinta floodplain generate a substantial economic value, which we estimate around $USD 5,000 million per year from a wetland area of 773,707 ha. The limitations of the simple value transfer methodology applied in this study include the inherent difficulties involved in international value transfer (Lindhjem & Navrud, 2008) and the potential overdue influence that individual value observations may have on the results (Pendleton et al., 2016). In spite of such limitations, the elicited values are meant to provide a useful basis for further investigation in primary valuation studies or for the application of more sophisticated value transfer techniques such as meta-regression (De Groot et al., 2012). Enlarging the number of new primary valuation studies available from Latin America and, more specifically, Mexico would be beneficial for future application of value transfer techniques as well as the design of protocols or guidelines concerning the information to be included in primary studies for value transfer purposes. In the context of the case study area, such further analyses are encouraged by the substantial size of the estimated ecosystem service values whose size corresponds to about half of the Gross Domestic Product in the entire state of Campeche (INEGI, 2014).

The study also reveals interesting differences and suggests potential complementarities between different valuation techniques. Although cultural and regulating services were highly valued in the benefit transfer exercise, provisioning services, especially food and support of commercial fishing, were identified as the most important services for local communities in the semi-structured interviews. Although the latter is commonly expected in the context of developing countries and regions, where food production (e.g., by fishing) sustains human well-being by meeting basic human needs (Béné, Hersoug & Allison, 2010; Daw et al., 2011), previous studies (Scholte, Van Teeffelen & Verburg, 2015; Martín-López et al., 2014) suggest that the difference with regulating services, which were highly valued in the benefit transfer exercise but had the lowest relative importance in the participatory interviews, may be at least partially imputed to the fact that ecosystem services that provide direct benefits are often more visible to local inhabitants who easily recognize how they depend on such services, while indirect benefits from cultural and regulating services are less tangible or require higher expertise on the side of the respondents to perceive them (Agbenyega et al., 2009). This suggest potential complementarities between the two approaches implemented in this study. Future studies may focus on providing a more comprehensive and representative social perception analysis of wetland ecosystem services by local communities by increasing the number of local communities and interviews, and including the perception of multiple stakeholders in order to understand their respective needs and priorities in the management of the wetlands (Iniesta-Arandia et al., 2014) and thus prevent tensions obstructing concerted action (Wilshusen, 2003). In addition, it is important to apply more inclusive approaches in order to evaluate local perceptions of conservation mechanisms implemented by governments in this important región, assuming that enviromental conservation will only be legitimate if local communities participate in management of natural resources and benefit from conservation (Wilshusen et al., 2002; Lele et al., 2010).

Overall, the results of the valuation components of the study are consistent with the notion that the ecosystem services provided by the Usumacinta floodplain have a significant flow of contributions to the well-being of local communities which is currently not included in the state’s accounts. Future land use changes altering these ecosystems could have a significant impact on the supply of ecosystem services, and therefore on the livelihoods of the local rural communities (Fisher & Christopher, 2007; Bouma, Joy & Steyn, 2013; Delgado & Marín, 2016; Ghermandi, Camacho-Valdez & Trejo-Espinosa, 2020). While we acknowledge that the proposed approach is a pragmatic rather than theory-driven one, which partly relies on secondary sources and combines data originally developed at different spatial scales, we believe it contributes to the development of the field insofar as: (1) it creates a bridge at the science-policy interface whereby it shows how widely used and readily recognized measures of ecosystem service values (i.e., monetary unit value transfer estimates from international repositories) can be combined with socio-cultural techniques and enriched with additional dimensions of analysis; and (2) it develops a rapid assessment tool which can be useful in providing policy-makers in data-poor countries or regions (such as, but not limited to, the investigated region in Mexico) with initial information on values and priority areas, which can be used as a starting basis for more in-depth, targeted investigation.

Integrating the ecosystem service valuation analysis into a broader perspective allows to infer further recommendations for the sustainable management of the local natural capital. The implemented spatial analysis shows which of the important zones for biodiversity are faced with deep marginalization and the ecosystem services that are most relevant in those areas, thus helping to define a first prioritization of areas for intervention or, if the opportunity arises for further and more in-depth investigation. This type of integration in a spatially explicit context, is crucial for identifying and communicating the relative importance of conservation activities in the context of limited availability of resources for ecosystem protection and could be applied to similar environments at regional, country or global level. Also, it may be useful in contexts where approximate value estimates and local perceptions are sufficient to greatly improve cost-benefit analyses for large scale developments, which can affect large areas of land, as in many locations around the world, when they are projected in the short term. For example, since 1956 the possibility of building a dam on the Usumacinta River has been proposed. The Federal Electricity Commission through its program ‘Works and Investments in the Electricity Sector’ (Comisión Federal de Electricidad, 2009) plans to complete the construction of Tenosique Dam Project (2017) in the Usumacinta Watershed. If the construction and operation of the dam materialize, the local effects might be seen in a loss of fishing productivity and bird diversity of the Centla Swamps and Terminos Lagoon, ecosystems of great importance in the Usumacinta floodplain (March y Castro 2003). As has been shown in other important river basins around the world (Winemiller et al., 2016), socio-ecological consequences of disrupting river flow are often underestimated in light of the potential regional benefits of hydrological energy.

Arguably, the Usumacinta floodplain wetlands are at high risk given that federal policy has concentrated on encouraging growth through increases in the Gross Domestic Product, with little consideration for other measures of well-being (Costanza et al., 2014), resulting, among others, in substantial changes in land cover over time (Ghermandi, Camacho-Valdez & Trejo-Espinosa, 2020). The present analysis shows that the livelihoods of people living in this region still strongly depend on the flow of ecosystem services provided by the Usumacinta floodplain, many of which are provided for “free” by nature and are thus not included in national accounting. To respond to such challenge, it is increasingly common for national government through environmental institutions and academia to perform national ecosystem services assessments to answer essential questions in relation to how ecosystem services are changing, where important services originate, and what should be protected and restored to increase the provision of ecosystem services (Ouyang et al., 2016).

## Conclusions

The results show that wetlands in the Usumacinta floodplain generate a significant economic estimated value; at least 4 billion USD was delivered to the inhabitants of this region in 2007, highlighting the palustrine ecosystems as the most important wetland in terms of covered area and value. Overall, cultural and provisioning services were the most relevant ecosystem services in terms of their monetary value and local perceived importance. Decision makers in this region will have to prioritize management policies in the municipality of Palizada, an area which coincides with the sites of the analysis of local perception, as well as where the three indicators in the spatial approach (ecosystem service values, marginalization and biodiversity) were highly significant.

The approach used in this study can provide a useful starting point for a more comprehensive analysis due to the ease with which the generated information and knowledge can be integrated into a more democratic policy design, and the broad scope of analysis which explicitly accounts for the perception of ecosystem services by local main users and allows for the internalization of the most important wetland ecosystem services in value analyses, with potential benefits in terms of the improvement of the livelihoods of the local communities.

##  Supplemental Information

10.7717/peerj.8395/supp-1 Supplemental Information 1 Raw data of value observations used in the ecosystem service analysis

## Additional Information and Declarations

### Field Study Permissions

The following information was supplied relating to field study approvals (i.e., approving body and any reference numbers): We did not require a formal field permit because during the project we did not collect any living specimens. All local and federal authorities were informed of our activities. The selection and contact with the communities during 2015-16 was carried out with the support of the Coordinator of the Natural Protected Area of Flora and Fauna ”Laguna de Terminos” José Guadalupe Carballo Reyes, area where the interview sites were located.

### Data Availability

The following information was supplied regarding data availability: The raw measurements are available as a Supplementary File.
