# Supplementary material for: Spatial analysis, local people’s perception and economic valuation of wetland ecosystem services in the Usumacinta floodplain, Southern Mexico
Source: PeerJ. 2020 Jan 31;8:e8395. doi: 10.7717/peerj.8395 (PMC6996512; doi:10.7717/peerj.8395)
Supplement: Supplemental Information 1 [file peerj-08-8395-s001.docx]

**Online Supporting Information**

This Appendix gives an overview of all 216 value observations used in the ecosystem service analysis. We provide an overview of the monetary values of ecosystem services per wetland, which include the number of used values, the mean, the standard deviation of the mean, the maximum and the minimum value. In addition for each value estimate by each wetland type we include the ecosystem service, the monetary value (in $US 2007 per year), the country/region, and original reference. All full references are listed by region at the end of this appendix. The design of the tables is based on the work by de Groot et al. (2012).

**A.1. Overview of used value per wetland type**

**1. Mangrove**

Description: forested-shrub estuarine wetland: plant association formed by one or a combination of the four species of mangrove.

Table 1.1. Ecosystem services values provided by mangroves (in $US 2007/ha/year).

| **MANGROVE** | No. obs. | Median value | S.D | Maximum value | Minimum value |
| --- | --- | --- | --- | --- | --- |
| **Provisioning services** | **72** | **1,325** | **11175** | **61864** | **684** |
| Support of commercial fishing | 30 | 380 | 10521 | 58628 | 0 |
| Water supply | 1 | 683 |  | 683 | 683 |
| Harvesting of natural materials | 23 | 213 | 321 | 1372 | 0 |
| Fuel Wood | 18 | 48 | 333 | 1181 | 0 |
| Ornamental resources |  |  |  |  |  |
| **Regulation services** | **10** | **690** | **2776** | **8095** | **7** |
| Local climate control | 1 | 7 |  | 7 | 7 |
| Flood control and storm buffering | 9 | 683 | 2776 | 8088 | 0 |
| Water quality improvement |  |  |  |  |  |
| **Support services** | **5** | **247** | **12347** | **27856** | **20** |
| Genetic resources |  |  |  |  |  |
| Biodiversity | 5 | 247 | 12347 | 27856 | 20 |
| **Cultural services** | **4** | **391** | **190** | **721** | **391** |
| Amenity and esthetics |  |  |  |  |  |
| Recreational activities | 3 | 362 | 190 | 692 | 362 |
| Recreational fishing and hunting | 1 | 28 |  | 28 | 28 |
| **Total** | **91** | **2,653** | **26489** | **98535** | **1101** |

Table 1.2. Monetary values per ecosystem service

| **Ecosystem services** | **Value** | **Country** | **Reference** | |
| --- | --- | --- | --- | --- |
| **Provisioning services** |  |  |  |  |
| Support of commercial fishing | 53.99 | Bangladesh | Ahmad | 1984 |
| Support of commercial fishing | 109.10 | Cambodia | Bann | 1997 |
| Support of commercial fishing | 51.08 | Cambodia | Bann | 1997 |
| Support of commercial fishing | 20.02 | Cambodia | Bann | 1997 |
| Support of commercial fishing | 240.98 | Mexico | Barbier | 1998 |
| Support of commercial fishing | 8829.90 | Malaysia | Bennett | 1993 |
| Support of commercial fishing | 316.09 | Indonesia | Burbridge | 1985 |
| Support of commercial fishing | 178.94 | Thailand | Christensen | 1982 |
| Support of commercial fishing | 596.46 | Thailand | Christensen | 1982 |
| Support of commercial fishing | 1228.71 | Thailand | Christensen | 1982 |
| Support of commercial fishing | 357.88 | Thailand | Christensen | 1982 |
| Support of commercial fishing | 72.02 | Malaysia | Dugan | 1990 |
| Support of commercial fishing | 942.35 | El Salvador | Gammage | 1997 |
| Support of commercial fishing | 432.68 | Trinidad and Tobago | Hamilton | 1984 |
| Support of commercial fishing | 686.05 | Fiji | Lal | 1990 |
| Support of commercial fishing | 944.82 | Vietnam | Levine | 1998 |
| Support of commercial fishing | 6215.62 | Australia | Morton | 1990 |
| Support of commercial fishing | 550.56 | Trinidad and Tobago | Ramdial | 1975 |
| Support of commercial fishing | 212.73 | Indonesia | Ruitenbeek | 1992 |
| Support of commercial fishing | 569.80 | Indonesia | Ruitenbeek | 1992 |
| Support of commercial fishing | 224.40 | Thailand | Sathirathai | 1998 |
| Support of commercial fishing | 379.97 | Thailand | Sathirathai | 1998 |
| Support of commercial fishing | 1951.6 | Malaysia | Bann | 1999 |
| Support of commercial fishing | 516.44 | Thailand | Barbier | 1996 |
| Support of commercial fishing | 352.90 | Costa Rica | IUCN | 2004 |
| Support of commercial fishing | 246.96 | El Salvador | MENR | 2002 |
| Support of commercial fishing | 58628.02 | Philippines | Nickerson | 1999 |
| Support of commercial fishing | 0.06 | Vietnam | Tri | 1996 |
| Support of commercial fishing | 16.04 | Vietnam | Tri | 2000 |
| Water supply | 683.45 | El Salvador | MENR | 2002 |
| Harvesting of natural materials | 53.99 | Bangladesh | Ahmad | 1984 |
| Harvesting of natural materials | 12.63 | Cambodia | Bann | 1997 |
| Harvesting of natural materials | 7.42 | Cambodia | Bann | 1997 |
| Harvesting of natural materials | 3.90 | Cambodia | Bann | 1997 |
| Harvesting of natural materials | 51.56 | Malaysia | Bennett | 1993 |
| Harvesting of natural materials | 79.02 | Indonesia | Burbridge | 1985 |
| Harvesting of natural materials | 1371.86 | Thailand | Christensen | 1982 |
| Harvesting of natural materials | 72.02 | Malaysia | Dugan | 1990 |
| Harvesting of natural materials | 10.48 | El Salvador | Gammage | 1997 |
| Harvesting of natural materials | 242.30 | Trinidad and Tobago | Hamilton | 1984 |
| Harvesting of natural materials | 2.54 | Pakistan | Khalil | 1999 |
| Harvesting of natural materials | 409.23 | Trinidad and Tobago | Ramdial | 1975 |
| Harvesting of natural materials | 212.73 | Indonesia | Ruitenbeek | 1992 |
| Harvesting of natural materials | 333.85 | Indonesia | Ruitenbeek | 1992 |
| Harvesting of natural materials | 305.61 | Indonesia | Ruitenbeek | 1992 |
| Harvesting of natural materials | 224.40 | Thailand | Sathirathai | 1998 |
| Harvesting of natural materials | 379.97 | Thailand | Sathirathai | 1998 |
| Harvesting of natural materials | 296.76 | El Salvador | MENR | 2002 |
| Harvesting of natural materials | 246.96 | El Salvador | MENR | 2002 |
| Harvesting of natural materials | 720.55 | Philippines | Nickerson | 1999 |
| Harvesting of natural materials | 0.06 | Vietnam | Tri | 1996 |
| Harvesting of natural materials | 20.78 | Vietnam | Tri | 2000 |
| Fuel Wood | 3.81 | Cambodia | Bann | 1997 |
| Fuel Wood | 3.91 | Cambodia | Bann | 1997 |
| Fuel Wood | 42.86 | Cambodia | Bann | 1997 |
| Fuel Wood | 2.34 | Cambodia | Bann | 1997 |
| Fuel Wood | 79.02 | Indonesia | Burbridge | 1985 |
| Fuel Wood | 1180.99 | Thailand | Christensen | 1982 |
| Fuel Wood | 72.02 | Malaysia | Dugan | 1990 |
| Fuel Wood | 10.48 | El Salvador | Gammage | 1997 |
| Fuel Wood | 22.32 | Pakistan | Khalil | 1999 |
| Fuel Wood | 23.21 | Fiji | Lal | 1990 |
| Fuel Wood | 409.23 | Trinidad and Tobago | Ramdial | 1975 |
| Fuel Wood | 212.73 | Indonesia | Ruitenbeek | 1992 |
| Fuel Wood | 296.76 | El Salvador | MENR | 2002 |
| Fuel Wood | 720.55 | Philippines | Nickerson | 1999 |
| Fuel Wood | 0.06 | Vietnam | Tri | 1996 |
| Fuel Wood | 1.89 | Vietnam | Tri | 2000 |
| **Regulation services** |  |  |  |  |
| Local climate control | 7 | Cambodia | Emerton | 2002 |
| Flood control and storm buffering | 74.68 | Indonesia | Ruitenbeek | 1992 |
| Flood control and storm buffering | 8087.79 | Thailand | Sathirathai | 1998 |
| Flood control and storm buffering | 3,131 | Malaysia | Bann | 1999 |
| Flood control and storm buffering | 5080.10 | Thailand | Barbier | 1996 |
| Flood control and storm buffering | 683.45 | El Salvador | MENR | 2002 |
| Flood control and storm buffering | 2005.00 | Philippines | Samonte | 2004 |
| Flood control and storm buffering | 448.00 | Cambodia | Emerton | 2002 |
| **Support services** |  |  |  |  |
| Biodiversity | 59.62 | Indonesia | Ruitenbeek | 1992 |
| Biodiversity | 27855.68 | Malaysia | Bann | 1999 |
| Biodiversity | 246.96 | El Salvador | MENR | 2002 |
| Biodiversity | 19.53 | Vietnam | Tri | 2000 |
| **Cultural services** |  |  |  |  |
| Recreational activities | 692.28 | Trinidad and Tobago | Hamilton | 1984 |
| Recreational activities | 362.28 | Trinidad and Tobago | Ramdial | 1975 |
| Recreational activities | 362.49 | Trinidad and Tobago | Ramdial | 1975 |
| Recreational fishing and hunting | 28.42 | Trinidad and Tobago | Ramdial | 1975 |

**2. Coastal lagoon**

Description: subtidal estuarine wetland.

Table 1.1. Ecosystem services values provided by coastal lagoon (in $US 2007/ha/year)

| **Coastal lagoon** | No. obs. | Median value | S.D | Maximum value | Minimum value |
| --- | --- | --- | --- | --- | --- |
| **Provisioning services** | **7** | **281** | **1093** | **2046** | **45** |
| Support of commercial fishing | 3 | 122 | 435 | 826 | 31 |
| Water supply | 1 | 3 |  | 3 | 3 |
| Harvesting of natural materials | 3 | 156 | 658 | 1217 | 11 |
| Fuel Wood |  |  |  |  |  |
| Ornamental resources |  |  |  |  |  |
| **Regulation services** | **3** | **17** | **208** | **370** | **4** |
| Local climate control |  |  |  |  |  |
| Flood control and storm buffering | 3 | 17 | 208 | 370 | 4 |
| Water quality improvement |  |  |  |  |  |
| **Support services** | **0** | **0** | **0** | **0** | **0** |
| Genetic resources |  |  |  |  |  |
| Biodiversity |  |  |  |  |  |
| **Cultural services** | **5** | **1629** | **4648** | **9175** | **700** |
| Amenity and esthetics | 1 | 460 |  | 460 | 460 |
| Recreational activities | 1 | 151 |  | 151 | 151 |
| Recreational fishing and hunting | 3 | 1018 | 4648 | 8565 | 89 |
| **Total** | **15** | **1,926** | **5949** | **11591** | **749** |

Table 1.2. Monetary values per ecosystem service

| **Ecosystem services** | **Value** | **Country** | **Reference** | |
| --- | --- | --- | --- | --- |
| **Provisioning services** |  |  |  |  |
| Support of commercial fishing | 30.76 | Italy | Nunes | 2004 |
| Support of commercial fishing | 825.87 | Trinidad and Tobago | Dharmaratne | 2002 |
| Support of commercial fishing | 122.09 | Cambodia | Emerton | 2002 |
| Water supply | 2.97 | US | Farber | 1996 |
| Harvesting of natural materials | 11.33 | Cambodia | Emerton | 2002 |
| Harvesting of natural materials | 155.80 | France | Rudloff | 1997 |
| Harvesting of natural materials | 1216.69 | France | Rudloff | 1997 |
| **Regulation services** |  |  |  |  |
| Flood control and storm buffering | 3.99 | US | Farber | 1987 |
| Flood control and storm buffering | 16.87 | US | Farber | 1996 |
| Flood control and storm buffering | 369.83 | US | Farber | 1996 |
| **Cultural services** |  |  |  |  |
| Amenity and esthetics | 459.77 | Morocco | Benessiah | 1998 |
| Recreational activities | 150.8 | Italy | Signorello | 1998 |
| Recreational fishing and hunting | 1018.07 | Morocco | Benessiah | 1998 |
| Recreational fishing and hunting | 89.47 | Italy | Alberini | 2007 |
| Recreational fishing and hunting | 8564.70 | France | Dabat | 1998 |

**3. Palustrine**

Description: palustrine continental wetland (where there is permanent water): swamp, marshes, tular, popal.

Table 1.1. Ecosystem services values provided by palustrine (in $US 2007/ha/year)

| **Palustrine** | No. obs. | Median value | S.D | Maximum value | Minimum value |
| --- | --- | --- | --- | --- | --- |
| **Provisioning services** | **19** | **4,845** | **6800** | **13687** | **519** |
| Support of commercial fishing | 9 | 67 | 1659 | 5056 | 11 |
| Water supply | 6 | 1286 | 867 | 2116 | 39 |
| Harvesting of natural materials | 2 | 3353 | 4275 | 6376 | 330 |
| Fuel Wood | 1 | 26 |  | 26 | 26 |
| Ornamental resources | 1 | 114 |  | 114 | 114 |
| **Regulation services** | **11** | **2322** | **8501** | **20831** | **308** |
| Local climate control |  |  |  |  |  |
| Flood control and storm buffering | 3 | 1661 | 2550 | 5200 | 250 |
| Water quality improvement | 8 | 661 | 5951 | 15631 | 58 |
| **Support services** | **4** | **55** | **277** | **528** | **40** |
| Genetic resources | 1 | 31 |  | 31 | 31 |
| Biodiversity | 3 | 24 | 277 | 497 | 9 |
| **Cultural services** | **5** | **2467** | **20867** | **43502** | **504** |
| Amenity and esthetics |  |  |  |  |  |
| Recreational activities | 4 | 2,430 | 20867 | 43465 | 468 |
| Recreational fishing and hunting | 1 | 37 |  | 37 | 37 |
| **Total** | **39** | **9,689** | **36446** | **78548** | **1372** |

Table 1.2. Monetary values per ecosystem service

| **Ecosystem services** | **Value** | **Country** | **Reference** | |
| --- | --- | --- | --- | --- |
| **Provisioning services** |  |  |  |  |
| Support of commercial fishing | 148.22 | US | Dillman | 1993 |
| Support of commercial fishing | 40.54 | Uganda | Emerton | 1998 |
| Support of commercial fishing | 10.94 | Sweden | Folke | 1991 |
| Support of commercial fishing | 54.28 | US | Leitch | 1996 |
| Support of commercial fishing | 36.64 | US | Leitch | 1996 |
| Support of commercial fishing | 221.17 | US | Leitch | 1996 |
| Support of commercial fishing | 93.63 | US | Leitch | 1996 |
| Support of commercial fishing | 5055.92 | Italy | Franco | 2006 |
| Support of commercial fishing | 67.35 | US | Milon | 1999 |
| Water supply | 577.64 | Uganda | Emerton | 1998 |
| Water supply | 38.70 | Sweden | Folke | 1991 |
| Water supply | 770.38 | Japan | Kuriyama | 1998 |
| Water supply | 1999.16 | Japan | Kuriyama | 1998 |
| Water supply | 2115.54 | Japan | Kuriyama | 1998 |
| Water supply | 1800.68 | Japan | Kuriyama | 1998 |
| Harvesting of natural materials | 330.01 | Uganda | Emerton | 1998 |
| Harvesting of natural materials | 6375.84 | Netherlands | Bos | 1998 |
| Fuel wood | 25.59 | US | Ko | 2004 |
| Ornamental resources | 114 | South Africa | Adekola | 2008 |
| **Regulation services** |  |  |  |  |
| Flood control and storm buffering | 250.48 | US | Blomquist | 1998 |
| Flood control and storm buffering | 1661.48 | US | Blomquist | 1998 |
| Flood control and storm buffering | 5055.92 | Italy | Franco | 2006 |
| Water quality improvement | 295.12 | US | Breaux | 1995 |
| Water quality improvement | 2932.58 | US | Breaux | 1995 |
| Water quality improvement | 15631.32 | US | Breaux | 1995 |
| Water quality improvement | 148.22 | US | Dillman | 1993 |
| Water quality improvement | 10672.27 | Uganda | Emerton | 1998 |
| Water quality improvement | 57.77 | Sweden | Folke | 1991 |
| Water quality improvement | 1026.20 | US | Cardoch | 2000 |
| Water quality improvement | 243.34 | US | Ko | 2004 |
| **Support services** |  |  |  |  |
| Genetic resources | 31 | Western Africa | Ly | 2006 |
| Biodiversity | 9.09 | Sweden | Folke | 1991 |
| Biodiversity | 23.80 | UK | Hanley | 1991 |
| Biodiversity | 496.83 | UK | Turner | 1988 |
| **Cultural services** |  |  |  |  |
| Recreational activities | 43465.17 | Netherlands | Bos | 1998 |
| Recreational activities | 2814.46 | US | Cooper | 1991 |
| Recreational activities | 467.82 | Italy | Marangon | 2002 |
| Recreational activities | 2045.93 | Italy | Marangon | 2002 |
| Recreational fishing and hunting | 36.64 | US | Leitch | 1996 |

**4. Riverine**

Description: permanent riverine wetland; rivers and channels

Table 1.1. Ecosystem services values provided by riverine (in $US 2007/ha/year)

| **Riverine** | No. obs. | Median value | S.D | Maximum value | Minimum value |
| --- | --- | --- | --- | --- | --- |
| **Provisioning services** | **29** | **1,085** | **16345** | **53726** | **186** |
| Support of commercial fishing | 11 | 822 | 12504 | 42542 | 2 |
| Water supply | 8 | 18 | 2476 | 6864 | 1 |
| Harvesting of natural materials | 9 | 65 | 1365 | 4140 | 3 |
| Fuel Wood | 1 | 180 |  | 180 | 180 |
| Ornamental resources |  |  |  |  |  |
| **Regulation services** | **22** | **4,399** | **119313** | **447449** | **3** |
| Local climate control |  |  |  |  |  |
| Flood control and storm buffering | 14 | 4,291 | 114963 | 434958 | 1 |
| Water quality improvement | 8 | 108 | 4350 | 12490 | 2 |
| **Support services** | **10** | **2,145** | **6162** | **20553** | **0** |
| Genetic resources |  |  |  |  |  |
| Biodiversity | 10 | 2,145 | 6162 | 20553 | 0 |
| **Cultural services** | **8** | **5,202** | **2024** | **7403** | **3418** |
| Amenity and esthetics | 1 | 3,291 |  | 3291 | 3291 |
| Recreational activities | 5 | 1,253 | 1150 | 2835 | 87 |
| Recreational fishing and hunting | 2 | 658 | 874 | 1277 | 40 |
| **Total** | **51** | **12,833** | **143845** | **529130** | **3607** |

Table 1.2. Monetary values per ecosystem service

| **Ecosystem services** | **Value** | **Country** | **Reference** | |
| --- | --- | --- | --- | --- |
| **Provisioning services** |  |  |  |  |
| Support of commercial fishing | 42541.93 | US | Mullarkey | 1997 |
| Support of commercial fishing | 102.54 | Angola | Seyam | 2001 |
| Support of commercial fishing | 91.75 | Angola | Seyam | 2001 |
| Support of commercial fishing | 1696.87 | US | Whitehead | 1991 |
| Support of commercial fishing | 3443.59 | Lao | Gerrard | 2004 |
| Support of commercial fishing | 1.54 | Bangladesh | Islam | 2006 |
| Support of commercial fishing | 6.84 | Cambodia | IUCN | 2005 |
| Support of commercial fishing | 821.94 | Cambodia | Navy | 2000 |
| Support of commercial fishing | 67.34 | Mozambique | Turpie | 1999 |
| Support of commercial fishing | 4139.62 | China | Tong | 2007 |
| Support of commercial fishing | 4146.89 | China | Tong | 2007 |
| Water supply | 6863.83 | France | ACSA | 1996 |
| Water supply | 2.98 | Bangladesh | Islam | 2006 |
| Water supply | 6.84 | Cambodia | IUCN | 2005 |
| Water supply | 8.70 | Mozambique | Turpie | 1999 |
| Water supply | 0.70 | Namibia, Botswana | Turpie | 1999 |
| Water supply | 27.79 | Mozambique | Turpie | 1999 |
| Water supply | 955.86 | China | Tong | 2007 |
| Water supply | 3187.12 | China | Tong | 2007 |
| Harvesting of natural materials | 64.76 | Angola | Seyam | 2001 |
| Harvesting of natural materials | 1276.63 | Czech Republic | Ungerman | 1994 |
| Harvesting of natural materials | 855.62 | Lao | Gerrard | 2004 |
| Harvesting of natural materials | 2.93 | Cambodia | IUCN | 2005 |
| Harvesting of natural materials | 13.78 | Cambodia | Navy | 2000 |
| Harvesting of natural materials | 3.42 | Angola | Seyam | 2001 |
| Harvesting of natural materials | 6.27 | Tanzania | Turpie | 2000 |
| Harvesting of natural materials | 69.11 | Mozambique | Turpie | 1999 |
| Harvesting of natural materials | 4139.62 | China | Tong | 2007 |
| Fuel wood | 180.45 | Cambodia | Navy | 2000 |
| **Regulation services** |  |  |  |  |
| Flood control and storm buffering | 46565.23 | US | Dalecki | 1993 |
| Flood control and storm buffering | 11947.59 | France | ACSA | 1996 |
| Flood control and storm buffering | 434958.22 | France | ACSA | 1996 |
| Flood control and storm buffering | 4314.41 | France | ACSA | 1996 |
| Flood control and storm buffering | 39.22 | France | ACSA | 1996 |
| Flood control and storm buffering | 6004.10 | Lao | Gerrard | 2004 |
| Flood control and storm buffering | 2428.09 | US | Leschine | 1997 |
| Flood control and storm buffering | 7356.56 | US | Leschine | 1997 |
| Flood control and storm buffering | 8536.73 | US | Leschine | 1997 |
| Flood control and storm buffering | 11.14 | US | Schultz | 2001 |
| Flood control and storm buffering | 0.67 | Mozambique | Turpie | 1999 |
| Flood control and storm buffering | 4.41 | Namibia, Botswana | Turpie | 1999 |
| Flood control and storm buffering | 1280.44 | China | Tong | 2007 |
| Flood control and storm buffering | 4268.33 | China | Tong | 2007 |
| Water quality improvement | 317.62 | Germany | Gren | 1995 |
| Water quality improvement | 12490.32 | France | ACSA | 1996 |
| Water quality improvement | 10.00 | France | ACSA | 1996 |
| Water quality improvement | 2745.53 | France | ACSA | 1996 |
| Water quality improvement | 148.07 | Lao | Gerrard | 2004 |
| Water quality improvement | 18.91 | Mozambique | Turpie | 1999 |
| Water quality improvement | 2.23 | Namibia, Botswana | Turpie | 1999 |
| Water quality improvement | 68.19 | Mozambique | Turpie | 1999 |
| **Support services** |  |  |  |  |
| Biodiversity | 20553.00 | Austria | Kosz | 1996 |
| Biodiversity | 2835.45 | Australia | Mallawaarachchi | 2001 |
| Biodiversity | 2446.00 | France | Amigues | 1998 |
| Biodiversity | 40.11 | Australia | Bennett | 2002 |
| Biodiversity | 3290.93 | UK | Garrod | 1994 |
| Biodiversity | 1844.60 | Germany | Meyerhoff | 2004 |
| Biodiversity | 0.09 | Angola | Seyam | 2001 |
| Biodiversity | 6099.26 | Germany | Hanusch | 2000 |
| Biodiversity | 1006.73 | Germany | Brauer | 2004 |
| Biodiversity | 250.11 | France | El Yousfi | 2006 |
| **Cultural services** |  |  |  |  |
| Amenity and esthetics | 3290.93 | UK | Garrod | 1994 |
| Recreational activities | 2835.45 | Australia | Mallawaarachchi | 2001 |
| Recreational activities | 1696.87 | US | Whitehead | 1991 |
| Recreational activities | 1253.14 | US | Azevedo | 2000 |
| Recreational activities | 87.10 | Italy | Marangon | 2002 |
| Recreational activities | 139.71 | Spain | Segui | 2004 |
| Recreational fishing and hunting | 1276.63 | Czech Republic | Ungerman | 1994 |
| Recreational fishing and hunting | 40.11 | Australia | Bennett | 2002 |

**5. Lacustrine**

Description: lacustrine continental wetland, permanent and seasonal; lake, ponds, body of water.

Table 1.1. Ecosystem services values provided by lacustrine (in $US 2007/ha/year)

| **Lacustrine** | No. obs. | Median value | S.D | Maximum value | Minimum value |
| --- | --- | --- | --- | --- | --- |
| **Provisioning services** | **8** | **478** | **1170** | **2610** | **31** |
| Support of commercial fishing | 4 | 446 | 1148 | 2563 | 16 |
| Water supply | 2 | 24 | 12 | 32 | 15 |
| Harvesting of natural materials | 2 | 8 | 10 | 15 | 0 |
| Fuel Wood |  |  |  |  |  |
| Ornamental resources |  |  |  |  |  |
| **Regulation services** | **1** | **23** | **0** | **23** | **23** |
| Local climate control |  |  |  |  |  |
| Flood control and storm buffering | 1 | 23 |  | 23 | 23 |
| Water quality improvement |  |  |  |  |  |
| **Support services** | **2** | **4,369** | **5933** | **8565** | **174** |
| Genetic resources |  |  |  |  |  |
| Biodiversity | 2 | 4,369 | 5933 | 8565 | 174 |
| **Cultural services** | **18** | **1,496** | **4721** | **13053** | **62** |
| Amenity and esthetics | 7 | 495 | 1538 | 4149 | 37 |
| Recreational activities | 7 | 896 | 3130 | 8772 | 12 |
| Recreational fishing and hunting | 4 | 105 | 53 | 132 | 14 |
| **Total** | **29** | 6,366 | **11825** | **24251** | **291** |

Table 1.2. Monetary values per ecosystem service

| **Ecosystem services** | **Value** | **Country** | **Reference** | |
| --- | --- | --- | --- | --- |
| **Provisioning services** |  |  |  |  |
| Support of commercial fishing | 431.43 | US | Amacher | 1989 |
| Support of commercial fishing | 461.37 | Malawi | Schuyt | 2002 |
| Support of commercial fishing | 15.76 | Malawi | Schuyt | 2002 |
| Support of commercial fishing | 2562.57 | Uganda | MacLean | 2003 |
| Water supply | 15.17 | Canada | Kooten | 1992 |
| Water supply | 31.96 | Malawi | Schuyt | 2002 |
| Harvesting of natural materials | 0.33 | Malawi | Schuyt | 2002 |
| Harvesting of natural materials | 15.12 | Canada | Vuuren | 1993 |
| **Regulation services** |  |  |  |  |
| Flood control and storm buffering | 23.46 | US | Hovde | 1994 |
| **Support services** |  |  |  |  |
| Biodiversity | 173.98 | France | Desaigues | 1991 |
| Biodiversity | 8564.70 | France | Dabat | 1998 |
| **Cultural services** |  |  |  |  |
| Amenity and esthetics | 388.26 | US | Carman | 1992 |
| Amenity and esthetics | 4149.46 | Australia | Gerrans | 1994 |
| Amenity and esthetics | 494.55 | Netherlands | Groot | 1998 |
| Amenity and esthetics | 1417.84 | Netherlands | Groot | 1998 |
| Amenity and esthetics | 36.65 | Canada | Kreutzwiser | 1981 |
| Amenity and esthetics | 2513.18 | Canada | Kreutzwiser | 1981 |
| Amenity and esthetics | 67.53 | US | Roberts | 1997 |
| Recreational activities | 11.66 | Thailand | Dixon | 1990 |
| Recreational activities | 28.22 | US | Hovde | 1994 |
| Recreational activities | 8771.98 | US | Azevedo | 2000 |
| Recreational activities | 1681.48 | Netherlands | Groot | 1998 |
| Recreational activities | 1520.56 | Kenya | Navrud | 1994 |
| Recreational activities | 895.98 | Kenya | Navrud | 1994 |
| Recreational activities | 86.99 | France | Desaigues | 1991 |

**A.2. Primary studies per continent**

**AFRICA**

Adekola, O., Morardet, S., De Groot, R., & Grelot, F. (2008, September). The economic and livelihood value of provisioning services of the Ga-Mampa wetland, South Africa. In 13th IWRA World Water Congress (pp. 24-p).

Benessaiah, N., & Belhaj, M. (1998). Mediterranean wetlands socioeconomic aspects. Ramsar Convention Bureau.

Emerton, L., Iyango, L., Luwum, P., & Malinga, A. (1999). The present economic value of Nakivubo urban wetland, Uganda. IUCN—The World Conservation Union, Eastern Africa Regional Office, Nairobi and National Wetlands Programme, Wetlands Inspectorate Division, Ministry of Water, Land and Enviornment, Kampala.

Ly, O. K., Bishop, J., Moran, D., & Dansokho, M. (2006). Estimating the value of ecotourism in the Djoudj National Bird Park in Senegal. IUCN.

Maclean, I. M., Tinch, R., Hassall, M., & Boar, R. R. (2003). Towards optimal use of tropical wetlands: an economic valuation of goods derived from papyrus swamps in southwest Uganda. Environmental Change and Management Working Paper, 2003, 10.

Navrud, S., & Mungatana, E. D. (1994). Environmental valuation in developing countries: the recreational value of wildlife viewing. Ecological Economics, 11(2), 135-151.

Schuijt, K. (2002). Land and water use of wetlands in Africa: Economic values of African wetlands.

Seyam, I. M., Hoekstra, A. Y., Ngabirano, G. S., & Savenije, H. H. G. (2001). The value of freshwater wetlands in the Zambezi basin. Value of water research report series, (7), 22.

Turpie, J., Smith, B., Emerton, L., & Barnes, J. (1999). Economic value of the Zambezi Basin Wetlands. IUCN Zambezi Basin Wetlands Conservation and Resource Utilization Project.

Turpie, J. K. (2000). The use and value of natural resources of the Rufiji floodplain and delta, Tanzania. Unpublished report to IUCN (EARO).

**ASIA**

Ahmad, N. (1984). Some aspects of economic resources of Sundarban mangrove forest of Bangladesh. In Asian Symposium on Mangrove Environment: Research and Management, Kuala Lumpur (Malaysia), 25-29 Aug 1980.

Bann, C. (1997). An economic analysis of alternative mangrove management strategies in Koh Kong Province, Cambodia. Economy and Environment Programme for Southeast Asia (EEPSEA).

Bann, C. (1999). A Contingent valuation of the mangroves of Benut, Johor State, Malaysia. Economy and Environment Programme for Southeast Asia (EEPSEA).

Barbier, E. B., Heal, G. M., & Geoffrey, M. (2006). *Valuing ecosystem services* (p. 2). Berkeley Electronic Press.

Bennett, E. L., & Reynolds, C. J. (1993). The value of a mangrove area in Sarawak. Biodiversity and Conservation, 2(4), 359-375.

Burbridge, P. R. (1985). Management of mangrove exploitation in Indonesia. In Asian Symposium on Mangrove Environment: Research and Management, Kuala Lumpur (Malaysia), 25-29 Aug 1980.

Christensen, B. O. (1982). Management and utilization of mangroves in Asia and the Pacific (No. 3).

Dixon, J. A., & Sherman, P. B. (1990). Economics of protected areas: a new look at benefits and costs. Island Press.

Dugan, P. J. (Ed.). (1990). Wetland conservation: A review of current issues and required action. IUCN.

Emerton, L., Seilava, R., & Pearith, H. (2002). Bokor, Kiriron, Kep and Ream National Parks, Cambodia: Case studies of Economic and Development Linkages. Review of Protected Areas and their Role in the Socio-economic Development of the Four Countries of the Lower Mekong Region. Karachi: International Centre for Environmental Management.

Emerton, L. (2005). Values and rewards: counting and capturing ecosystem water services for sustainable development (No. 1). IUCN.

Gerrard, P. (2004). Integrating wetland ecosystem values into urban planning: the case of that Luang Marsh. Vientiane, Lao PDR, WWF Lao PDR and IUCN–The World Conservation Regional Environmental Economics Programme Asia, Colombo.

Gürlük, S., & Rehber, E. (2008). A travel cost study to estimate recreational value for a bird refuge at Lake Manyas, Turkey. Journal of environmental management, 88(4), 1350-1360.

Islam, M., & Braden, J. B. (2006). Bio-economic development of floodplains: farming versus fishing in Bangladesh. Environment and Development Economics, 11(01), 95-126.

Khalil, S. (1999). Economic valuation of the mangrove ecosystem along the Karachi coastal areas. The Economic Value of the Environment: Cases from South Asia. Published by IUCN.

Kuriyama, K. (1998). Measuring the value of the ecosystem in the Kushiro wetland: an empirical study of choice experiments. Forest Economics and Policy Working Paper, 9802.

Levine, S., & Mindedal, M. (1998). Economics of Multiple-Use Natural Resources: The Mangroves of Vietnam (MSc Thesis, Copenague University).

Navy, H., Somony, T., & Viseth, H. (1993). Valuation of Flooded Forests in Kandal Province, Cambodia. Economy and Environment: Case Studies in Cambodia. Economy and Environment Programme for Southeast Asia (EEPSEA): Singapore.

Nickerson, D. J. (1999). Trade-offs of mangrove area development in the Philippines. Ecological Economics, 28(2), 279-298.

Ruitenbeek, H. J. 1992. Mangrove management: An economic analysis of management options with a focus on Bintuni Bay, Irian Jaya. Menteri Negara Kependudukan dan Lingkungan Hidup.

Samonte-Tan, G. P., White, A. T., Tercero, M. A., Diviva, J., Tabara, E., & Caballes, C. (2007). Economic valuation of coastal and marine resources: Bohol Marine Triangle, Philippines. Coastal Management, 35(2-3), 319-338.

Sathirathai, S. (1998). Economic valuation of mangroves and the roles of local communities in the conservation of natural resources: case study of Surat Thani, South of Thailand. South Bridge: Economy and Environment Program for Southeast Asia.

Tong, C., Feagin, R. A., Lu, J., Zhang, X., Zhu, X., Wang, W., & He, W. (2007). Ecosystem service values and restoration in the urban Sanyang wetland of Wenzhou, China. Ecological engineering, 29(3), 249-258.

Tri, N. H., Adger, N., Kelly, M., Granich, S., & Ninh, N. H. (1996). The role of natural resource management in mitigating climate impacts: mangrove restoration in Vietnam. Center for Social and Economic Research on the Global Environment (CSERGE). Working Paper GEC, 96-06.

Tri, N. H., Hong, P. N., Manh, M. N. T., Tuan, M. L. X., Anh, M. P. H., Tho, M. N. H., ... & Tuan, M. L. D. (2000). Valuation of the Mangrove Ecosystem in Can Gio Mangrove Biosphere Reserve, Vietnam. UNESCO/MAB Project, Final Report.

**AUSTRALASIA**

Gerrans, P. (1994). An economic valuation of the Jandakot wetlands. Edith Cowan University occasional paper 1.

Lal, P. N. (1990). Conservation or conversion of mangroves in Fiji: an ecological economic analysis. East-West Centre Occasional Papers 11.

Mallawaarachchi, T., Blamey, R. K., Morrison, M. D., Johnson, A. K. L., & Bennett, J. W. (2001). Community values for environmental protection in a cane farming catchment in Northern Australia: A choice modelling study. Journal of Environmental Management, 62(3), 301-316.

Morton, R. M. (1990). Community structure, density and standing crop of fishes in a subtropical Australian mangrove area. Marine Biology, 105(3), 385-394.

Whitten, S. M., & Bennett, J. W. (2001). Private and social values of wetlands research reports. School of Economics and Management, University College, The University of South Wales, Canberra.

**EUROPE**

Alberini, A., Zanatta, V., & Rosato, P. (2007). Combining actual and contingent behavior to estimate the value of sports fishing in the Lagoon of Venice. Ecological Economics, 61(2), 530-541.

Amigues, J. P., & Desaigues, B. (1999). L'évaluation d'une politique de protection de la biodiversité des forëts riveraines de la Garonne. La valeur économique des hydrosystèmes. Méthodes et modèles d'évaluation des services délivrés, Point, P (éd.), Paris: Economica, 37, 62.

Birol, E., Karousakis, K., & Koundouri, P. (2006). Using a choice experiment to account for preference heterogeneity in wetland attributes: the case of Cheimaditida wetland in Greece. Ecological economics, 60(1), 145-156.

Birol, E., Karousakis, K., & Koundouri, P. (2006). Using economic valuation techniques to inform water resources management: A survey and critical appraisal of available techniques and an application. Science of the total environment, 365(1), 105-122.

Bos, E. J., & van den Bergh, J. C. (1998). Economic Evaluation, Land/Water Use, and Sustainable Nature Conservation of'De Vechtstreek'Wetlands (No. 98-036/3). Tinbergen Institute Discussion Paper.

Bräuer, I. (2005). Valuation of ecosystem services provided by biodiversity conservation: an integrated hydrological and economic model to value the enhanced nitrogen retention in renaturated streams. In Valuation and conservation of biodiversity (pp. 193-204). Springer Berlin Heidelberg.

Bureau d'Etudes Acsa. (1996). Evaluation économique des services rendus par les zones humides. S. Aoubid, & H. Gaubert (Eds.). Commissariat général au développement durable, Service de l'économie, de l'évaluation et de l'intégration du développement durable.

Dabat, M. H., & Rudloff, M. A. (1999). La valeur de préservation d'une lagune méditerranéenne menacée de comblement. La valeur économique des hydrosystèmes: méthodes et modèles d'évaluation des services délivrés. Ed. Point.

De Groot, R. S. (1993). Environmental functions and the economic value of natural ecosystems. In: Jansson, A. (Eds). Investing in natural capital: the ecological economics approach to sustainability. Island Press.

El Yousfi, H., Nicolaï, S., & Casin, P. (2006). Etude économique sur les coûts et bénéfices environnementaux dans le domaine de l'eau: l'île de Rhinau. Agence de l'Eau Rhin-Meuse, Université Paul Verlaine de Metz, Rapport de stage.

Franco, D., Mannino, I., Piccioni, E., Favero, L., Mattiuzzo, E., & Zanetto, G. (2006). Stima del valore economico totale delle zone umide in Veneto. Estimo e Territorio, 9, 32-48.

Folke, C. (1991). The societal value of wetland life-support. In Linking the Natural Environment and the Economy: Essays from the Eco-Eco Group (pp. 141-171). Springer Netherlands.

Garrod, G. D., Willis, K. G., & Saunders, C. M. (1994). The benefits and costs of the Somerset Levels and Moors ESA. Journal of rural studies, 10(2), 131-145.

Gren, M., & Söderqvist, T. (1994). Economic valuation of wetlands: a survey (No. 54). Beijer International Institute of Ecological Economics, The Royal Swedish Academy of Sciences.

Gren, M., Groth, K. H., & Sylvén, M. (1995). Economic values of Danube floodplains. Journal of Environmental Management, 45(4), 333-345.

Hanley, N., & Craig, S. (1991). Wilderness development decisions and the Krutilla-Fisher model: the case of Scotland's ‘flow country’. Ecological Economics, 4(2), 145-164.

Hanusch, H., Cantner, U., & Muench, K. (2000). Erfassung und Bewertung der Umweltwirkungen des Ausbaus der Donaustrecke Straubing-Vilshofen.

Kosz, M. (1996). Valuing riverside wetlands: the case of the “Donau-Auen” national park. Ecological Economics, 16(2), 109-127.

Ledoux, L. (2003, March). Wetland valuation: state of the art and opportunities for further development. In Proceedings of a Workshop Organised for the Environment Agency by Environmental Futures Ltd. and CSERGE. Bristol: Environment Agency (pp. 5-17).

Marangon, F., Tempesta, T., & Visintin, F. (2002, October). Turismo e attività ricreative nelle aree protette italiane: un quadro conoscitivo ancora inadeguato. In 2nd Conferenza Nazionale delle Aree Naturali Protette (pp. 11-13).

Meyerhoff, J., & Dehnhardt, A. (2004). The European Water Framework Directive and economic valuation of wetlands. In Proc. of 6th BIOECON Conference Cambridge.

Nunes, P. A., Rossetto, L., & de Blaeij, A. (2004). Measuring the economic value of alternative clam fishing management practices in the Venice Lagoon: results from a conjoint valuation application. Journal of Marine Systems, 51(1), 309-320.

Rudloff, M. A., Salles, J. M., & Boisson, J. M. (1997). Evaluation monétaire du patrimoine naturel: pratiques et enjeux pour les espaces littoraux: application à l'étang de Thau (Master thesis. University of Montpellier).

Seguí Amórtegui, L. A. (2004). *Sistemas de regeneración y reutilización de aguas residuales. Metodología para el análisis técnico-económico y casos*. Universitat Politècnica de Catalunya.

Signorello, G. (1998). Valuing birdwatching in a Mediterranean wetland. In Environmental Resource Valuation (pp. 173-191). Springer US.

Turner, R. K., & Brooke, J. (1988). Management and valuation of an environmentally sensitive area: Norfolk Broadland, England, case study. Environmental Management, 12(2), 193-207.

**NORTH AMERICA**

Amacher, G. S., Brazee, R. J., Bulkley, J. W., & Moll, R. A. (1989). Application of wetland valuation techniques: Examples from great lakes coastal wetlands (No. PB-90-112319/XAB). Michigan State Univ., East Lansing, MI (USA). Inst. Of Water Research.

Azevedo, C. D., Herriges Sr, J. A., & Kling, C. (2000). Iowa wetlands: Perceptions and values. Iowa State University, Center for Agricultural and Rural Development, Staff Report 00-SR 91

Barbier, E. B., & Strand, I. (1998). Valuing mangrove-fishery linkages–a case study of Campeche, Mexico. Environmental and Resource Economics, 12(2), 151-166.

Blomquist, G. C., & Whitehead, J. C. (1991). Measuring contingent values for wetlands: effects of information about related environmental goods. Water Resource Research, 2.

Blomquist, G. C., & Whitehead, J. C. (1998). Resource quality information and validity of willingness to pay in contingent valuation. Resource and Energy Economics, 20(2), 179-196.

Breaux, A., Farber, S., & Day, J. (1995). Using natural coastal wetlands systems for wastewater treatment: an economic benefit analysis. Journal of environmental management, 44(3), 285-291.

Cardoch, L., Day, J. W., Rybczyk, J. M., & Kemp, G. P. (2000). An economic analysis of using wetlands for treatment of shrimp processing wastewater—a case study in Dulac, LA. Ecological Economics, 33(1), 93-101.

Carman, M., Lamb, G., Miller, A., Sadowske, S., & Shaffer, R. (1992). The Oconto Waterfront: Issues and Options: A Survey of Oconto Residents. University of Wisconsin–Extension/Madison, Center for Community Economic Development.

Cooper, J., & Loomis, J. (1991). Economic value of wildlife resources in the San Joaquin Valley: hunting and viewing values. In The Economics and Management of Water and Drainage in Agriculture (pp. 447-462). Springer US.

Dalecki, M. G., Whitehead, J. C., & Blomquist, G. C. (1993). Sample non-response bias and aggregate benefits in contingent valuation: an examination of early, late and non-respondents. Journal of Environmental Management, 38(2), 133-143.

Dillman, B. L., Beran, L. J., & Hook, D. D. (1993). Nonmarket Valuation of Freshwater Wetlands: The Francis Beidler Forest. South Carolina Water Resources Research Institute, Clemson University.

Farber, S. (1987). The value of coastal wetlands for protection of property against hurricane wind damage. Journal of Environmental Economics and Management, 14(2), 143-151.

Farber, S. (1996). Welfare loss of wetlands disintegration: A Louisiana study. Contemporary Economic Policy, 14(1), 92-106.

Hovde, B., & Leitch, J. A. (1994). Valuing prairie potholes: Five case studies. Department of Agricultural Economics, Agricultural Experiment Station, North Dakota State University.

Johnson, C. W., & Linder, R. L. (1986). An economic valuation of South Dakota wetlands as a recreation resource for resident hunters. Landscape Journal, 5(1), 33-38.

Ko, J. Y., Day, J. W., Lane, R. R., & Day, J. N. (2004). A comparative evaluation of money-based and energy-based cost–benefit analyses of tertiary municipal wastewater treatment using forested wetlands vs. sand filtration in Louisiana. Ecological Economics, 49(3), 331-347.

Kreutzwiser, R. (1981). The economic significance of the Long Point marsh, Lake Erie, as a recreational resource. Journal of Great Lakes Research, 7(2), 105-110.

Kreutzwiser, R. (1981). Recreational values of lakeshore marshes. In Proceedings of the Ontario Wetlands Conference: Hosted by the Federation of Ontario Naturalists and the Department of Applied Geography, Ryerson Polytechnical Institute, September 18-19, Toronto, ON (pp. 48-57).

Leitch, J. A., & Hovde, B. (1996). Empirical valuation of prairie potholes: Five case studies. Great Plains Research, 25-39.

Leschine, T. M., Wellman, K. F., & Green, T. H. (1997). The economic value of wetlands: wetlands' role in flood protection in Western Washington. Washington State Department of Ecology.

Milon, J. W., Hodges, A. W., Rimal, A., Kiker, C. F., & Casey, F. (1999). Public preferences and economic values for restoration of the Everglades/South Florida ecosystem. Economics Report, 99, 1.

Mullarkey, D. J. (1997). Contingent valuation of wetlands: testing sensitivity to scope (Doctoral dissertation, University of Wisconsin, Madison).

Raphael, C. N., & Jaworski, E. (1979). Economic value of fish, wildlife, and recreation in Michigan's coastal wetlands. Coastal Management, 5(3), 181-194.

Roberts, L. A., & Leitch, J. A. (1997). Economic valuation of some wetland outputs of Mud Lake, Minnesota-South Dakota. Agricultural Economics Report No.381, Department of Agricultural Economics, North Dakota State University.

Schultz, S. D., & Leitch, J. A. (2001). The feasibility of wetland restoration to reduce flooding in the Red River Valley: a case study of the Maple River Watershed, North Dakota. Department of Agribusiness and Applied Economics, Agricultural Experiment Station, North Dakota State University.

Van Vuuren, W., & Roy, P. (1993). Private and social returns from wetland preservation versus those from wetland conversion to agriculture. Ecological Economics, 8(3), 289-305.

Whitehead, J. C., Groothuis, P. A., Southwick, R., & Foster-Turley, P. (2006). Economic Values of Saginaw Bay Coastal Marshes. Department of Economics, Appalachian State University. Working Paper 06-10

**SOUTH AND CENTRAL AMERICA**

Carranza, A. (2007). Valoración económica del humedal Barrancones, municipio de Pasaquina, departamento de la Unión, El Salvador. International Union for Conservation of Nature and Natural Resources (2007). Valoración Económica Ecológica y Ambiental: Análisis de casos en Iberoamérica. Costa Rica. Editorial Universidad Nacional de Heredia, 604p, 375-406.

Dharmaratne, G., & Strand, I. (2002). Adaptation to climate change in the Caribbean: the role of economic valuation. Report to the Caribbean Planning for Adaptation to Climate Change Project, London.

Gammage, S. (1997). Estimating the returns to mangrove conversion: sustainable management or short term gain?. IIED Environmental Economics Programme Discussion Paper

Hamilton, L. S., & Snedaker, S. C. (1984). Handbook for mangrove area management. Honolulu: East-West Environment and Policy Institute.

Ramdial, B. S. (1975). The social and economic importance of the caroni swamp in Trinidad and Tabago. PhD Thesis, University of Michigan.

IUCN. (2004). Economic valuation study of the Térraba-Sierpe wetlands. IUCN Report.
